# Effects of prenatal exercise interventions on maternal body composition: A secondary analysis of the FitMum randomized controlled trial

**DOI:** 10.1371/journal.pone.0308214

**Published:** 2024-08-01

**Authors:** Caroline Borup Roland, Parisa Seyedhosseini, Signe de Place Knudsen, Anne Dsane Jessen, Ida Karoline Bach Jensen, Jane M. Bendix, Gerrit van Hall, Stig Molsted, Saud Abdulaziz Alomairah, Ellen Løkkegaard, Bente Stallknecht, Tine D. Clausen

**Affiliations:** 1 Department of Biomedical Sciences, University of Copenhagen, Copenhagen, Denmark; 2 Department of Gynaecology and Obstetrics, Copenhagen University Hospital–North Zealand, Hilleroed, Denmark; 3 Department of Nuclear Medicine and Clinical Physiology, Copenhagen University Hospital–North Zealand, Hilleroed, Denmark; 4 Department of Clinical Research, Copenhagen University Hospital–North Zealand, Hilleroed, Denmark; 5 Clinical Biochemistry, Clinical Metabolomics Core Facility, Rigshospitalet, Copenhagen, Denmark; 6 Department of Clinical Medicine, University of Copenhagen, Copenhagen, Denmark; 7 Department of Mental Health, John Hopkins Bloomberg School of Public Health, Baltimore, MD, United States of America; 8 Department of Gynecology, Fertility and Obstetrics, Copenhagen University Hospital–Rigshospitalet, Copenhagen, Denmark; McMaster University, CANADA

## Abstract

The main objective of the study was to investigate the effects of prenatal exercise interventions on maternal body composition at 28 weeks gestation and 7–14 days after delivery. We also explored associations between physical activity (PA) *per se* and body composition. This study presents secondary outcomes of the FitMum randomized controlled trial, which included healthy inactive pregnant women at gestational age ≤ 15+0 weeks. They were randomized to structured supervised exercise training, motivational counselling on PA, or standard care. Maternal body composition was measured by doubly labeled water at 28 weeks gestation (*n* = 134) and by dual-energy X-ray absorptiometry scan 7–14 days after delivery (*n* = 117). PA, including moderate-to-vigorous-intensity PA (MVPA), active kilocalories, and steps, were measured continuously from inclusion to delivery by a wrist-worn activity tracker. One hundred fifty pregnant women were included with a median pre-pregnancy body mass index (BMI) of 24.1 (21.6–27.9) kg/m^2^. We found no differences between groups in fat mass, fat percentage or fat-free mass at 28 weeks gestation or 7–14 days after delivery. Visceral adipose tissue mass and bone mineral density measured 7–14 days after delivery did not differ between groups either. Linear regression analyses adjusted for pre-pregnancy BMI showed that a higher number of daily steps was associated with lower fat mass, fat percentage, and visceral adipose tissue mass at 28 weeks gestation and 7–14 days after delivery. Active kilocalories during pregnancy was positively associated with fat-free mass 7–14 days after delivery. Neither structured supervised exercise training nor motivational counselling on PA during pregnancy affected maternal body composition at 28 weeks gestation or 7–14 days after delivery compared to standard care. Interestingly, when adjusted for pre-pregnancy BMI, higher number of daily steps was associated with lower fat content during pregnancy and after delivery, whereas MVPA and active kilocalories were not.

**Trial registration:** ClinicalTrials.gov; NCT03679130; 20/09/2018.

## Introduction

Maternal obesity and excessive gestational weight gain (GWG) pose global challenges, increasing the risk of adverse pregnancy outcomes and long-term obesity and cardiometabolic diseases for both mother and child [1–3]. Measuring maternal body weight, body mass index (BMI) and GWG reflect the total changes in several maternal and fetal components such as fat mass, fat-free mass, total body water and placenta, but total body weight measurements do not reveal the precise contribution of each component [4]. While fat mass is expected to increase to some extend during pregnancy [4–6] due to expansion of subcutaneous fat etc., excessive fat mass seems to be associated with impaired maternal cardiometabolic health during pregnancy [6–9]. Previous studies have shown that fat mass, fat mass index (fat mass divided by height squared) and fat percentage are strongly associated with insulin resistance during pregnancy [6–9], gestational diabetes mellitus, and other markers of cardiovascular disease [7]. Maternal body composition during all trimesters of pregnancy has also been associated with short-term infant health outcomes including birth weight [10–13], early postpartum infant fat mass [14, 15], and fat-free mass [15]. Moreover, two small observational studies have indicated that postpartum maternal BMI and body composition may be associated with breast milk composition and might hereby play a role in childhood obesity [16, 17]. Thus, understanding changes in maternal body composition during pregnancy and postpartum in the absence of clinically significant change in GWG may be of interest.

Prenatal physical activity (PA) measured by different methods has been indicated by some studies to be associated with changes in maternal body composition measures [18–20]. However, only few randomized controlled trials have been conducted in this field and these were either targeted overweight women or used inferior methods to measure body composition [21, 22]. An accurate assessment of maternal body composition seems important to gain a more detailed understanding of how changes in volume and distribution of different tissues relate to maternal and infant metabolic health. Advanced methods can be used for assessing body composition during pregnancy, such as the doubly labeled water (DLW) technique, but major limitations include the inability to distinguish between maternal and fetal tissues, as well as regional distribution [23–25]. Dual-energy X-ray absorptiometry (DXA) is the most widely used method to measure body composition in women who are not pregnant and men. DXA shows similar accuracy as magnetic resonance imaging, which (together with computed tomography) is considered the *gold standard* for measurement of body composition [26]. The use of DXA scans are not recommended during pregnancy due to ionizing radiation exposure [25], but DXA measurements of body composition just before and/or after pregnancy have been suggested to be meaningful for obtaining insights into maternal body composition during pregnancy [23].

The aim of the present randomized controlled study was to investigate the effects of prenatal exercise interventions on maternal body composition measured by DLW at 28 weeks gestation and by DXA scans 7–14 days after delivery. Additionally, we explored the associations between measures of PA *per se* (from randomization to 28+6 weeks gestation and to delivery, respectively) and various measures of body composition at 28 weeks gestation as well as 7–14 days after delivery.

## Materials and methods

### Participants and study design

The FitMum study was a randomized controlled trial conducted in 2018–2021 at the Department of Gynecology and Obstetrics at Copenhagen University Hospital–North Zealand, Hilleroed, Denmark (recruitment from October 1^st^ 2018 to October 15^th^ 2020). Healthy (no pre-existing or ongoing obstetric or medical complications), inactive (structured exercise at moderate-to-vigorous intensity < 1 hour/week during early pregnancy) women with gestational age ≤ 15+0 weeks were eligible for inclusion. This paper reports secondary outcomes of the study and includes participants with available data from DLW analysis (*n* = 134) and/or DXA scan (*n* = 117). We have previously published a detailed description of the study protocol [27] and the results of the primary outcome of the study, moderate-to-vigorous-intensity PA (MVPA) level [28]. Demographic information was obtained at inclusion and obstetric (e.g., gestational diabetes mellitus and gestational hypertensive disorders) and neonatal outcomes (birth weight and birth length) were collected from medical records. Pre-pregnancy BMI (kg/m^2^) was calculated based on self-reported pre-pregnancy weight and height. GWG at 40+0 weeks gestation was estimated based on predicted body weights from a mixed effects model as previously described [29] and excessive GWG was defined according to the Institute of Medicine’s recommendations [4]. Small for gestational age (< 10th percentile) and large for gestational age (> 90th percentile) [30] were defined according to a Scandinavian reference population and calculated using the Marsal formula [31], which includes fetal sex, birth weight and gestational age at delivery.

Written informed consent was obtained from all participants. The study was approved by the Danish National Committee on Health Research Ethics (August 30, 2018, #H-18011067) and the Danish Data Protection Agency (September 12, 2018, #P-2019-512). The study adheres to the principles of the Helsinki declaration.

### Randomization, interventions, and physical activity measures

Randomization in a 1:2:2 ratio to standard care (CON), structured supervised exercise training (EXE), or motivational counselling on PA (MOT) occurred after a one-week baseline period (at latest 16+0 weeks gestation). Participants in the EXE intervention were offered one-hour supervised exercise training at moderate intensity three times per week in a gym and swimming pool. In the MOT intervention, participants were offered three group and four individual PA motivational counselling sessions of 1–2 hours duration during pregnancy and received a weekly personalized text message to increase PA level. Instructors with a bachelor’s or master’s degree in physiotherapy, exercise physiology or similar conducted the intervention activities. During the COVID19-pandemic (from March 11^th^, 2020, and throughout the intervention period) EXE and MOT sessions were conducted online using Zoom Cloud Meetings. The EXE group had access to the swimming pool for three months during this period. In a period, test visits were also conducted online and no DLW administration nor DXA scans were performed [27].

PA, including MVPA (min per week), active kilocalories (per day) and steps (per day), were measured continuously from inclusion to delivery by a wrist-worn activity tracker (Garmin Vivosport). Missing PA data due to non-wear time were imputed by multiple imputations in 25 data sets using a prespecified seed, preselected baseline variables (body weight, age, PA, education level, and parity), and the random forest imputation model from the mice R package [28, 32]. PA data used for association analyses included the average imputed values from randomization to 28+6 weeks gestation for analyses with body composition assessed by DLW at 28 weeks gestation, and from randomization to delivery for analyses with body composition assessed by DXA 7–14 days after delivery.

### Body composition measurements

#### Doubly labeled water

The doubly labeled water technique (DLW) can be used to estimate fat mass based on total body water. In the present study, DLW (Sercon Limited, UK) was orally administered (0.1 g of 99.98% D_2_O and 1.6 g of 10% ^18^O per kg body weight) at a test visit at 28 weeks gestation after the collection of two baseline urine samples. In the weeks following DLW intake, participants collected urine samples in the morning (not the first void) after 1, 4, 7, 11, and 14 days. Samples were immediately stored in the participant’s own freezer at around -18°C and transported to the hospital for storage at -80°C as soon as possible after day 14. Samples were shipped to the Clinical Metabolomics Core Facility at Rigshospitalet, Copenhagen, for analysis, which is described in detail by Alomairah et al. [33]. The total body water was calculated as the average of H_2_ and ^18^O dilution space divided by correction factors for *in vivo* isotopic exchange (1.04 for H_2_ and 1.01 for ^18^O) [34]. Total fat mass (kg) and fat-free mass (kg) at 28 weeks gestation were calculated from total body water and body weight at DLW administration day, based on equations for no edema or leg edema only [35] and specified to a gestational age of 28 weeks [23]. Fat-free mass is composed of water, protein, and bone minerals. Fat percentage (%) at 28 weeks gestation was calculated from the total fat mass and body weight at DLW administration day.

#### Dual-energy X-ray absorptiometry scans

Whole-body composition was measured by DXA scans (Hologic Discovery A/Horizon A, Marlborough, MA, US (*n* = 115), and GE Healthcare Lunar iDXA, Chicago, IL, US (*n* = 2)) 7–14 days after delivery at Department of Nuclear Medicine and Clinical Physiology, Copenhagen University Hospital–North Zealand, Hilleroed by trained staff blinded for study group allocation. Body composition measures included fat mass (kg), fat percentage (%), fat-free mass (kg), visceral adipose tissue mass (g), and bone mineral density (g/cm^2^). Fat-free mass was composed of the sum of lean mass and bone mineral content assessed by DXA.

### Statistical analyses

Data are presented as mean and standard deviation for approximately symmetric distributions, median and interquartile range for asymmetric distributions, and frequency and proportion for categorical data. Estimated effect sizes are presented as means with 95% confidence interval [95% CI]. Statistical analyses were performed using R (version 4.1.0) [36] and GraphPad Prism (version 9, GraphPad Software) and statistical significance was defined as a *p*-value < 0.05. Residuals were checked for normal distribution by visual inspection. One-way ANOVA was used to compare fat mass, fat percentage and fat-free mass (DLW and DXA), as well as visceral adipose tissue mass and bone mineral density (DXA) between the three study groups. Associations between PA measures *per se* and body composition measures were performed using linear regression with adjustment for pre-pregnancy BMI. Additionally, we performed linear regression analysis to explore the association between maternal pre-pregnancy BMI and MVPA, active kilocalories, as well as steps in the baseline period.

## Results

### Participant characteristics

**Fig 1:**
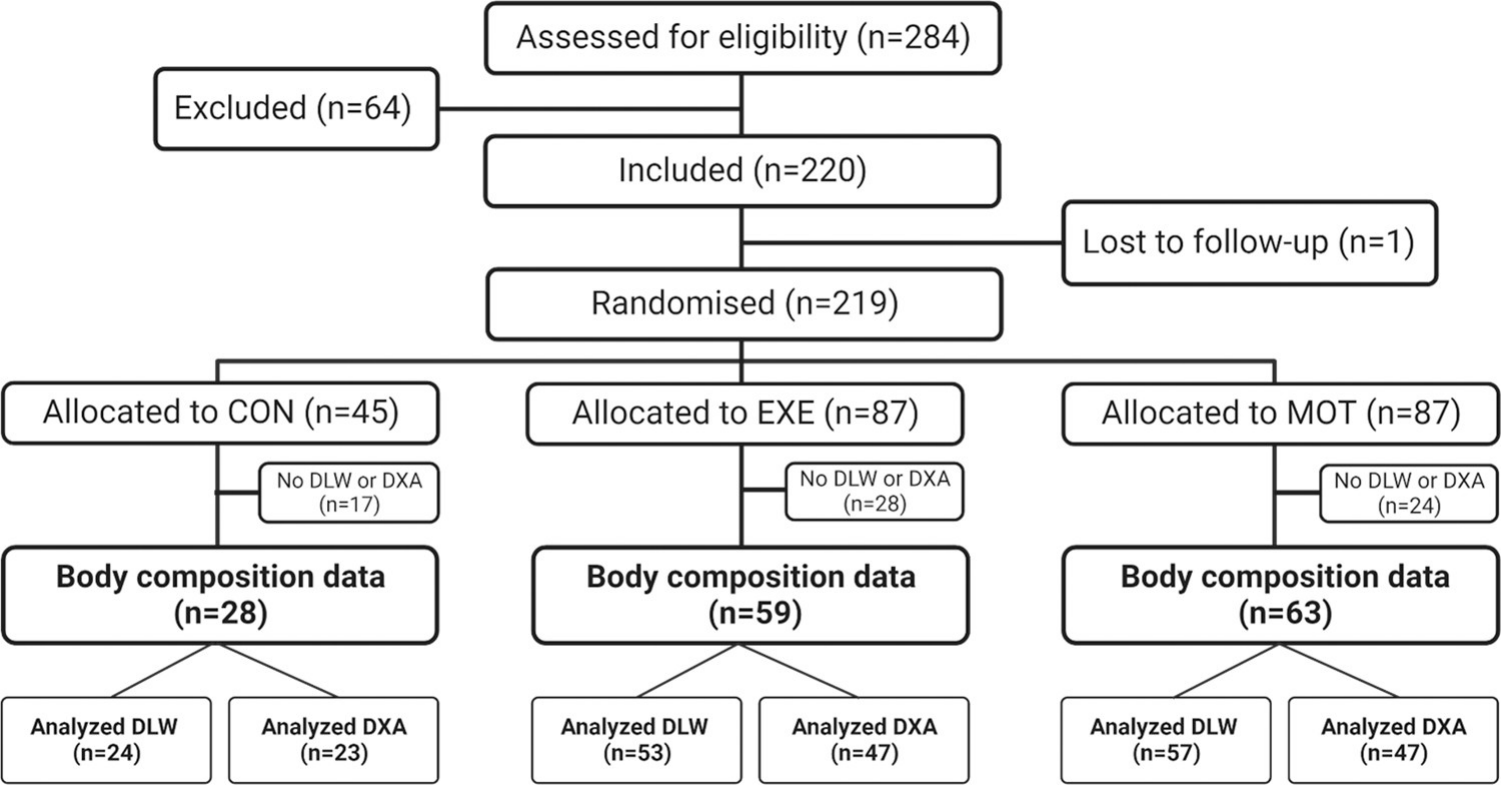
Inclusion, randomization, allocation, and analyzed body composition data in the FitMum study. Body composition data were obtained from 150 participants in total (CON: n = 28, EXE: n = 59, MOT: n = 63). Urine samples for DLW analyses were obtained from n = 24 in CON, n = 53 in EXE and n = 57 in MOT at gestational age 28 weeks. DXA scans carried out 7–14 days after delivery were available from n = 23 in CON, n = 47 in EXE and n = 47 in MOT. Both DLW and DXA data were available from 101 participants. CON; Control, DLW; Doubly labeled water, DXA; Dual-energy X-ray absorptiometry, EXE; Structured supervised exercise training, MOT; Motivational counselling on physical activity. The figure was created with BioRender.com.

The FitMum study included 220 women (219 randomized) and we obtained body composition data from in total 150 participants (CON: *n* = 28, EXE: *n* = 59, MOT: *n* = 63). Urine samples for DLW analyses were available from 134 participants (CON: *n* = 24, EXE: *n* = 53, MOT: *n* = 57) and DXA scans were available from 117 participants (CON: *n* = 23, EXE: *n* = 47, MOT: *n* = 47) (Fig 1).

We obtained both DLW and DXA data from 101 out of the 150 participants, whereas only DLW or DXA data was obtained from the remaining 49 participants. Descriptive characteristics of the 150 participants included in the present secondary analyses are shown in Table 1. Most participants were healthy and without obstetric complications, but more than 50% had an excessive GWG according to the Institute of Medicine’s recommendations [4]. Most offspring were delivered full term and had normal anthropometric measures. Descriptive characteristics seemed to be similar between the three study groups except for proportion of nulliparous participants, excessive GWG, infant sex, and proportion of children born either small or large for gestational age. No statistical comparisons have been performed on descriptive characteristics in accordance with CONSORT recommendations.

**Table 1:** Descriptive characteristics.

	ALL (n = 150)	CON (n = 28)	EXE (n = 59)	MOT (n = 63)
Age, years	31.4 (4.1)	31.1 (3.6)	30.8 (4.4)	32.0 (4.0)
Pre-pregnancy BMI, kg/m^2^*	24.1 (21.6–27.9)	23.3 (20.9–25.4)	24.2 (21.7–28.1)	24.3 (22.3–28.9)
Gestational age at inclusion, weeks	12.4 (9.6–13.7)	12.7 (9.9–13.5)	12.1 (9.5–13.3)	12.6 (9.7–14.0)
Parity, n (%)				
Nulliparous	59 (39)	9 (32)	29 (49)	21 (33)
Education level, n (%)				
School ≥12 years	137 (91)	26 (93)	51 (86)	60 (95)
Further education ≥3 years	130 (87)	23 (82)	52 (88)	55 (87)
Employment status, n (%)				
Employed/studying	137 (91)	26 (93)	55 (93)	56 (89)
Smoking, n (%)				
During pregnancy	0 (0)	0 (0)	0 (0)	0 (0)
Quitted smoking in relation to this pregnancy	14 (9)	4 (14)	6 (10)	4 (6)
Obstetric outcomes*				
Gestational diabetes mellitus, n (%)	10 (7)	1 (4)	5 (9)	4 (6)
Gestational hypertensive disorders, n (%)	10 (7)	2 (7)	3 (5)	5 (8)
GWG until 40+0 weeks gestation, kg	15.1 (5.7)	14.8 (4.6)	15.8 (4.9)	14.6 (6.9)
Excessive GWG, n (%)	86 (57)	15 (54)	39 (66)	32 (51)
Gestational age at delivery, weeks	40.3 (39.5–41.1)	40.1 (38.9–41.2)	40.6 (39.9–41.3)	40.0 (39.3–40.9)
Preterm birth (<37 weeks gestation), n (%)	4 (3)	2 (7)	0 (0)	2 (3)
Neonatal outcomes*				
Infant sex, n (%) males	69 (46)	10 (36)	34 (58)	25 (40)
Birth weight, g	3680 (3260–3920)	3580 (3070–3880)	3720 (3410–3990)	3660 (3180–3870)
Birth length, cm	52.0 (51.0–53.0)	52.0 (51.0–53.0)	52.0 (51.0–53.8)	52.0 (50.0–53.0)
Small for gestational age, n (%)	13 (9)	3 (11)	2 (3)	8 (13)
Large for gestational age, n (%)	10 (7)	1 (4)	2 (3)	7 (11)

Descriptive characteristics in the randomization groups. School ≥12 years corresponds to high school and further education ≥3 years corresponds to university degree (bachelor or master level). Gestational hypertensive disorders are defined as preeclampsia, gestational hypertension, HELLP (hemolysis, elevated liver enzymes, and low platelet count) syndrome or eclampsia.

*For some variables the total number is lower due to missing values: Pre-pregnancy BMI; n = 149 (CON; n = 28, EXE; n = 58, MOT; n = 63), Obstetric outcomes; n = 147 (CON; n = 28, EXE; n = 58, MOT; n = 61), Birth length; n = 146 (CON; n = 28, EXE; n = 58, MOT; n = 60), all other neonatal outcomes; n = 147 (CON; n = 28, EXE; n = 58, MOT; n = 61). Continuous variables are presented as mean (standard deviation) or median (interquartile range) and categorical variables are presented as n (%). CON; Control group, EXE; Structured supervised exercise training, GWG; Gestational weight gain, MOT; Motivational counselling on physical activity.

The descriptive characteristics and the distribution of participants in the three groups for the participants included in the present secondary analyses (*n* = 150) did not differ compared to the original sample of participants in the study (*n* = 219) [29]. Among the participants included in the present analyses, the median weekly MVPA from randomization to delivery was 32.5 minutes (min) (21.0–46.2) in CON, 47.1 min (27.5–92.3) in EXE and 31.8 min (21.7–56.8) in MOT. Among the same participants, adherence to EXE and MOT was on average 1.5 exercise sessions/week [1.3;1.7] out of the recommended 3 exercise sessions/week and 6.1 counselling sessions [5.7;6.5] out of the recommended 7 counselling sessions during pregnancy, respectively.

### Body composition at 28 weeks gestation and 7–14 days after delivery

We found no difference between the three study groups in total fat mass, fat percentage or fat-free mass as measured by DLW at 28 weeks gestation or by DXA scans 7–14 days after delivery (Table 2). Also, DXA scan measurements 7–14 days after delivery showed no differences between groups in visceral adipose tissue mass and bone mineral density (Table 2).

**Table 2:** Body composition at 28 weeks gestation and 7–14 days after delivery.

	CON	EXE	MOT	*p* ^§^
**Measures by DLW at 28 weeks gestation**	**n = 24**	**n = 53**	**n = 57**	
Fat mass (kg)	25.7 [22.3;29.0]	27.8 [24.8;30.8]	28.8 [26.3;31.3]	0.430
Fat percentage (%)	32.6 [30.4;34.8]	32.8 [30.8;34.9]	33.9 [32.2;35.6]	0.606
Fat-free mass (kg)	51.6 [49.2;53.9]	54.2 [52.6;55.9]	54.3 [52.7;55.9]	0.140
**Measures by DXA at 7–14 days after delivery**	**n = 23**	**n = 47**	**n = 47**	
Fat mass (kg)	27.6 [24.3;31.0]	29.9 [26.8;32.9]	30.5 [27.8–33.3]	0.483
Fat percentage (%)	35.5 [33.1;37.9]	36.9 [34.8;38.9]	36.6 [34.7;38.6]	0.693
Fat-free mass (kg)	49.4 [46.3;52.5]	49.3 [47.0;51.7]	51.7 [49.1;54.2]	0.326
Visceral adipose tissue mass (g)*	337 [262;412]	356 [308;405]	421 [355;487]	0.144
Bone mineral density (g/cm^2^)	1.14 [1.10;1.18]	1.11 [1.09;1.14]	1.16 [1.13;1.19]	0.055

Body composition measures in the randomization groups reported at 28 weeks gestation for participants with DLW data (n = 134) (CON: n = 24; EXE: n = 53; MOT: n = 57) and at 7–14 days after delivery for participants with DXA data (n = 117) (CON: n = 23; EXE: n = 47; MOT: n = 47).

*Visceral adipose tissue mass is obtained for 115 participants (CON: n = 22; EXE: n = 47; MOT: n = 46). Data are mean [95% confidence interval]. ^§^ANOVA was used to test difference between study groups. CON; Control, EXE; Structured supervised exercise training, MOT; Motivational counselling on physical activity.

### Pre-pregnancy body mass index and baseline physical activity

Maternal pre-pregnancy BMI was positively associated with both MVPA (*p* = 0.006) and active kilocalories (*p* < 0.001) at baseline, whereas no association was found between pre-pregnancy BMI and steps. Thus, we only report correlations between PA measures *per se* and body composition measures with adjustment for pre-pregnancy BMI.

### Associations between prenatal physical activity and body composition at 28 weeks gestation

Linear regression analyses adjusted for pre-pregnancy BMI showed no associations of MVPA with fat mass, fat percentage, or fat-free mass measured by DLW at 28 weeks gestation (Table 3). Similarly, no associations were found between active kilocalories and fat mass, fat percentage, or fat-free mass (Table 3). In contrast, higher number of steps was associated with lower fat mass (*p* = 0.026) and fat percentage (*p* = 0.007), but not with fat-free mass (Table 3).

**Table 3:** Associations between prenatal physical activity *per se* (from randomization to 28+6 weeks gestation) and body composition assessed by doubly labeled water at 28 weeks gestation, adjusted for pre-pregnancy body mass index.

	MVPA divided by 1000		Active kilocalories divided by 1000		Steps divided by 1000	
	*Slope [95% CI]*	*p*	*Slope [95% CI]*	*p*	*Slope [95% CI]*	*p*
Fat mass (kg)	-10.6 [-25.3;4.2]	0.159	-0.9 [-5.7;3.9]	0.705	-0.5 [-0.9;-0.1]	0.026
Fat percentage (%)	-10.6 [-24.9;3.7]	0.146	-2.9 [-7.6;1.7]	0.210	-0.6 [-1.0;-0.2]	0.007
Fat-free mass (kg)	-5.1 [-21.6;11.4]	0.544	3.1 [-2.2;8.4]	0.255	0.2 [-0.3;0.7]	0.515

N = 134. Physical activity measures were the average imputed values from randomization to 28+6 weeks of gestation and body composition measures were assessed by doubly labeled water at 28 weeks gestation. Linear regression analyses were performed with adjustment for pre-pregnancy BMI. MVPA; moderate-to-vigorous-intensity physical activity, CI; confidence interval

### Associations between prenatal physical activity and body composition 7–14 days after delivery

Linear regression analyses adjusted for pre-pregnancy BMI showed no associations between MVPA and fat mass, fat percentage, fat-free mass, visceral adipose tissue, or bone mineral density assessed by DXA 7–14 days after delivery (Table 4). A positive association was found between active kilocalories and fat-free mass (*p* = 0.022), but active kilocalories was not associated with other body composition measures (Table 4). In contrast, steps were negatively associated with fat mass (*p* = 0.002), fat percentage (*p* = 0.004), and visceral adipose tissue mass (*p* = 0.004). Steps were not associated with fat-free mass or bone mineral density (Table 4).

**Table 4:** Associations between prenatal physical activity *per se* (from randomization to delivery) and body composition assessed by dual-energy X-ray absorptiometry scan 7–14 days after delivery, adjusted for pre-pregnancy body mass index.

	MVPA divided by 1000		Active kilocalories divided by 1000		Steps divided by 1000	
	*Slope [95% CI]*	*p*	*Slope [95% CI]*	*p*	*Slope [95% CI]*	*p*
Fat mass (kg)	-3.0 [-20.0;14.1]	0.731	-1.2 [-6.0;3.7]	0.635	-0.9 [-1.4;-0.3]	0.002
Fat percentage (%)	-1.6 [-18.2;14.9]	0.846	-3.5[-8.2;1.2]	0.141	-0.8 [-1.3;-0.3]	0.004
Fat-free mass (kg)	-3.0 [-23.0;16.9]	0.763	6.5 [0.9;12.0]	0.022	0.3 [-0.4;0.9]	0.457
Visceral adipose tissue mass (g)	-144.3 [-653.7;365.0]	0.576	-11.1 [-159.7;137.5]	0.883	-25.4 [-42.4;-8.5]	0.004
Bone mineral density (g/cm^2^)	-0.2 [-0.5;0.1]	0.265	-0.1 [-0.2;0.01]	0.075	-0.0 [-0.01;0.01]	0.548

N = 117 for associations with fat mass, fat percentage, fat-free mass and bone mineral density. N = 115 for associations with visceral adipose tissue mass. Physical activity measures were the average imputed values from randomization to delivery and body composition measures were assessed by dual-energy X-ray absorptiometry scan at 7–14 days after delivery. Linear regression analyses were performed with adjustment for pre-pregnancy BMI. MVPA; moderate-to-vigorous-intensity physical activity, CI; confidence interval

## Discussion

In the present study we found no effects of EXE or MOT during pregnancy on fat mass, fat percentage or fat-free mass at 28 weeks gestation and at 7–14 days after delivery compared to CON. Visceral adipose tissue mass and bone mineral density at 7–14 days after delivery did not differ between groups either. Our findings from linear regression analyses adjusted for pre-pregnancy BMI showed that a higher number of steps *per se* was associated with lower fat mass, fat percentage, and visceral adipose tissue mass at 28 weeks gestation and 7–14 days after delivery, whereas MVPA and active kilocalories were not. Active kilocalories during pregnancy was however positively associated with fat-free mass 7–14 days after delivery. This association was not observed in relation to the other measures of PA.

Fat mass, fat percentage, and fat-free mass from both DLW and DXA analyses were similar in our and other studies investigating maternal body composition and PA in pregnant women with normal weight [19, 21]. It is noteworthy that our interventions did not improve body composition measures compared to CON. These results are in contrast to some non-randomized controlled studies in pregnant women indicating that higher PA is associated with lower fat mass [18–20]. On the other hand, in line with our results, two previous randomized controlled trials investigating effects of prenatal exercise interventions on maternal body composition showed no effects on fat mass, fat percentage or fat-free mass [21, 22] compared to control groups. The prenatal exercise interventions conducted in these other studies are somewhat comparable to the EXE intervention in the present study, since they consisted of 3–5 exercise sessions per week for 15–50 minutes and the studies included women with normal weight [21] and overweight and obesity [22].

The lack of intervention effects on body composition in the present study might be explained by the low adherence rate and low MVPA level among participants in EXE and MOT, since it is important to achieve a certain amount of PA to obtain beneficial health effects [37, 38]. In both EXE and MOT, the median weekly MVPA level from randomization to delivery was below 60 minutes per week and thus markedly lower than the recommended PA level by the Danish Health Authorities of 210 minutes per week at moderate intensity [37]. The COVID-19 pandemic started during the study conduction period but did not seem to affect PA level negatively in our overall study population (*n* = 219). For example MVPA did not differ between participants included before the COVID-19 pandemic (physical intervention only) and during the COVID-19 pandemic (online intervention only) in any of the three study groups [28]. Nevertheless, although the PA level among participants in our study was relatively low, it was likely better than what could be expected if the interventions were applied at a population level since we recognize that our study sample is healthy, has a low BMI, no chronic disease indications and might possibly be more motivated to be physically active than women who did not consent for the study.

Another reason for the negative findings in the present study could be that the study might be underpowered to detect differences. The numbers of participants in our study groups (CON: *n* = 28, EXE: *n* = 59, MOT: *n* = 63) were only slightly higher compared to the aforementioned randomized controlled trials by Cavalcante *et al*. [21] and Seneviratne *et al*. [22], who found no effects of prenatal exercise training on maternal body composition. Thus, even if assuming that our interventions would be effective on improving maternal body composition had adherence rate and MVPA level been higher, our study might be underpowered to detect effects on maternal fat mass, fat-free mass etc.

Moreover, we have not collected baseline data on maternal body composition specifically, but our data on maternal pre-pregnancy BMI reveal that we included women with relatively normal weight in our study, which might have reduced the potential for exercise to improve body composition compared to for example pregnant populations with overweight and obesity. Conversely, more than 50% of our study participants had an excessive GWG according to the Institute of Medicine’s recommendations [4], which indicates that there could be room for improvement of maternal body composition.

Interestingly, in line with previous studies investigating PA and fat mass in pregnant women [18–20], we found that higher number of daily steps was associated with lower fat content at 28 weeks gestation and 7–14 days after delivery, after adjusting for pre-pregnancy BMI. This is particularly interesting because walking seems to be the preferred PA modality among pregnant women, and it is unique compared to other PA modalities in that it may be more meaningfully integrated into some transportation and occupational activities [39]. In addition, Renault *et al*. [40] investigated a PA intervention including encouragement to increase number of daily steps in pregnant women with obesity, and found a lower GWG after the PA intervention compared to standard care. Obviously, we cannot conclude causality from our linear regression analysis results. The negative associations between steps and fat measures can express that participants with lower fat content took more daily steps.

Moreover, we found that a higher amount of active kilocalories during pregnancy was associated with higher fat-free mass 7–14 days after delivery. Previously, fat-free mass has also been shown to increase after exercise training in non-pregnant women [41], but to our knowledge, the present study is the first to show that higher prenatal PA in the form of active kilocalories is associated with higher fat-free mass in the early postpartum period.

### Strengths and limitations

A strength of the present study was that the effects of prenatal exercise interventions on maternal body composition were investigated in a randomized controlled design obtaining body composition data from a relatively large number of participants.

Moreover, body composition was measured both during pregnancy using DLW and early postpartum by DXA scan. DXA scan is considered a highly accurate method of measuring body composition, and despite this method being unapplicable during pregnancy due to radiation exposure, measurements immediately before conception or after delivery are still considered highly valuable for understanding body composition changes across pregnancy [23, 26]. Further, the use of DLW to assess body composition during pregnancy was a strength of the present study. When using DLW to assess body composition, total body water is measured and used to estimate fat mass and fat-free mass based on assumptions of the hydration of fat-free mass, calculated as the ratio between total body water and fat-free mass. A disadvantage of using DLW during pregnancy to estimate body composition measures is the need to assume the individual hydration of fat-free mass, since fat-free mass hydration changes during pregnancy, which may lead to errors in the body fat estimates [23, 25].

As expected, we found that higher maternal pre-pregnancy BMI was associated with higher MVPA and active kilocalories at baseline. When evaluating associations between PA and body composition in the present study, it was a limitation that the underlying algorithms for activity estimates provided from the commercial Garmin activity tracker are unavailable for researchers. For example, it was unclear how maternal body weight was handled in the algorithm. We assume that the PA estimates derived from the activity tracker were based on the participant’s heart rate measurements, as well as maternal body weight and other data entered in the associated Garmin Connect app, as described previously [33]. We did not find any association between pre-pregnancy BMI and number of daily steps at baseline. Thus, steps might be a more robust measure of PA across different BMI ranges, which could be explained by an assumption that number of daily steps is estimated based on the accelerometer technology in the activity tracker to a higher degree compared to MVPA and active kilocalories.

## Conclusions

Neither structured supervised exercise training nor motivational counselling on PA influenced maternal body composition at 28 weeks gestation or 7–14 days after delivery compared to standard care. Interestingly, when adjusted for pre-pregnancy BMI, higher number of daily steps during pregnancy was associated with lower fat content at 28 weeks gestation and 7–14 days after delivery, whereas MVPA and active kilocalories were not. Active kilocalories during pregnancy was however positively associated with fat-free mass 7–14 days after delivery. Low adherence to the interventions and low MVPA level in our study could account for the lack of positive effects of our interventions on maternal body composition outcomes. Future research is needed on novel strategies to improve adherence to PA during pregnancy and maybe even prior to conception.

## Supporting information

S1 Checklist CONSORT 2010 checklist of information to include when reporting a randomised trial*. (DOC)

S1 File (PDF)

S2 File (PDF)

## List of abbreviations

BMI: Body mass index
CON: Standard care
DLW: Doubly labeled water
DXA: Dual-energy X-ray absorptiometry
EXE: Structured supervised exercise training
GWG: Gestational weight gain
MOT: Motivational counselling on physical activity
MVPA: Moderate-to-vigorous-intensity physical activity
PA: Physical activity
