# Supplementary material for: Effects of prenatal exercise interventions on maternal body composition: A secondary analysis of the FitMum randomized controlled trial
Source: PLoS One. 2024 Aug 1;19(8):e0308214. doi: 10.1371/journal.pone.0308214 (PMC11293652; doi:10.1371/journal.pone.0308214)
Supplement: S1 File — (PDF) [file pone.0308214.s002.pdf]

Ellen Christine Leth Løkkegaard
Nordsjællands Hospital
Dyrehavevej 29
Gynækologisk Obstetrisk Afdeling
3400 Hillerød

Center for Sundhed

Regionsgården
Kongens Vænge 2
3400 Hillerød
Entrance B + D
Direct phone +45 38666329
Email annika.jacobsen@regionh.dk

Journal-nr.:H-18011067
Date: 30-08-2018

H-18011067 - FitMum RCT

Final approval.

The decision was made in accordance with Act No. 593 of 14 June 2011 on scientific ethical treatment of health and medical scientific research projects with later changes (combined in legislative decree no. 1083 of 15/09/2017).

I confirm receipt of the emails of 15 August 2018 and 21 August 2018, in response to the decision of 7 August 2018, in which conditions were set for the approval of the project.

The conditions for approval are deemed to have been met. The project has thus been finally approved.

The approval is valid until 31 December 2021 and includes the following documents:

• Trial protocol, version 5, of 18 July 2018

• Participant information, version 6, of 15 August 2018

• Participant information for guardians, version 5, dated 18 July 2018

• Informed consent form for project participants, version 3, of 9 May 2018

• Informed consent form for guardians, version 4, dated 13 June 2018

• Declaration of consent to receive information (recruitment), version 3, dated 9 May 2018

• Recruitment text for website

• Recruitment post and poster

• Leaflet

• The questionnaire approved for use in the trial: PSQI version 15 August 2018, PPAQ-DK version 15 August 2018, Prescreening Questionnaire version 15 August 2018, SF-36 version 15 August 2018, Sickness absence and or report version 15 August 2018, Pain and discomfort from lower back and pelvis version 15 August 2018, BREQ-2 version 15 August 2018, P-ESES-DK version 15 August 2018,

Furthermore, the following conditions apply to this project.

New bioinformatic data analyzes of data produced in the project must be reported to the committee that has approved the project.

Sequencing data cannot be used in a new research project without the committee's renewed permission. This applies both when used for a new purpose and when passed on to a third party. Note that the Danish Data Protection Authority must simultaneously allow disclosure to a third party, cf. § 10, subsection of the Personal Data Act. 3.

The approval applies to the notified trial sites and the notified principal investigator in Denmark.

Implementation of the project in violation of the approval can be punished with a fine or imprisonment, cf. Section 41 of the Committee Act.

Changes

If significant changes are made to the protocol material during the implementation of the project, these must be notified to the committee in the form of additional protocols. The changes may only be implemented after approval by the committee, cf. section 27, subsection of the committee act. 1.

Notification of additional protocols must be done electronically at www.drvk.dk/anmeldelse with the already assigned notification number and password.

Significant changes include changes that may have an impact on the safety of the subjects, interpretation of the scientific documentation on which the project is based and the implementation or management of the project. These can be, for example, changes in inclusion and exclusion criteria, trial design, number of trial subjects, trial procedures, duration of treatment, effect parameters, changes to those responsible for the trial or trial locations, as well as content changes in the written information material for the trial subjects.

Where new information means that the researcher is considering changing the procedure or stopping the trial, the committee must be informed of this.

Side effects and events

Ongoing reporting

The committee must be notified immediately if suspected serious, unexpected side effects or serious incidents occur during the project, cf. section 30, subsection of the committee act. 1. The report must be accompanied by comments on any consequences for the trial. Only side effects and incidents occurring in Denmark must be reported. Notification must be made no later than 7 days after the sponsor or the principal investigator has become aware of the incident.

When reporting, a form available at www.nvk.dk can be used. The form with attachments can be submitted electronically using a digital signature.

Annual report

Once a year during the entire trial period, the committee must have sent a list of all suspected serious (expected and unexpected) side effects and serious incidents that have occurred during the trial period together with a report on the safety of the test subjects, cf. section 30, subsection of the Committee Act. 2.

The material must be in Danish or English.

When reporting, a form found at www.nvk.dk must be used. The form with attachments can be submitted electronically using a digital signature.

Closing

The principal investigator and, if relevant, a sponsor must notify the committee of the completion of the project no later than 90 days after the end of the project, cf. section 31, subsection of the committee act. 1. The project is considered completed when the last subject has finished.

If the project is terminated earlier than planned, a reason for this must be sent to the committee no later than 15 days after the decision has been made, cf. section 31, subsection of the committee act. 2.

If the project is not started, this and the reason for this must be communicated to the committee.

The committee requests a copy of the final research report or publication, cf. Section 28, subsection of the Committee Act. 2. In relation to this, we must draw attention to the fact that there is an obligation to publish both negative, positive and inconclusive test results, cf. Section 20, subsection of the Committees Act. 1, No. 8.

The responsibility to report the final trial and report is shared between the principal investigator and, if relevant, a sponsor.

Oversight

The committee supervises that the project is carried out in accordance with the approval, cf. sections 28 and 29 of the committee act.

Signature on the declaration of consent

The committee draws attention to the fact that the principal investigator can delegate his/her duty to sign the informed consent form to the person holding the oral information interview. In that case, there must be a written delegation to this effect at the trial site.

Best regards
Mette S. Kjær
Chairman, Committee C
