# Supplementary material for: Effects of prenatal exercise interventions on maternal body composition: A secondary analysis of the FitMum randomized controlled trial
Source: PLoS One. 2024 Aug 1;19(8):e0308214. doi: 10.1371/journal.pone.0308214 (PMC11293652; doi:10.1371/journal.pone.0308214)
Supplement: S2 File — (PDF) [file pone.0308214.s003.pdf]

Clinical Trial Protocol

Trial ID: FitMum RCT

Title of Trial

FitMum randomized controlled trial

Principal Investigator:	Ellen Christine Leth Løkkegaard
Ethical Committee number:	61462
Clinicaltrials.gov number:	NCT03679130
Datatilsyn number:	VD-2018-336
Date:	18-08-2023
Version:	13

CONFIDENTIAL

Signatures:

**Principal Investigator:**	**Name**	Ellen Christine Leth Løkkegaard
	**Title**	Professor, chief physician, PhD
	**Department**	Department of Gynecology and Obstetrics, Nordsjælland's Hospital
	**Address:**	Dyrehavevej 29, DK-3400 Hillerød
	**Telephone:**	+45 4829 6249
	**E-mail:**	Ellen.christine.leth.loekkegaard@regionh.dk
	It is hereby declared that the trial will be performed according to the latest approved trial protocol and local regulatory requirements and legislation.	
	Signature	Date: 08-mar-2018
**Protocol Author:**	**Name**	Caroline Borup Roland
	**Title**	PhD Fellow
	**Department**	Department of Biomedical Sciences
	**Address:**	Blegdamsvej 3, DK-2200 Copenhagen N
	**Telephone:**	+45 4294 2065
	**E-mail:**	cba@sund.ku.dk
	Signature	Date: 8/3-2018

CONFIDENTIAL

Table of Contents

List of Abbreviations ... 5
1. Protocol Summary ... 6
2. Flowchart ... 8
3. Background / Rationale ... 9
 3.1 Trial rationale ... 9
 3.2 Benefit-risk assessments and ethical considerations ... 14
4. Objectives and endpoints ... 15
 4.1 Primary objective and hypotheses ... 15
 4.2 Secondary objectives ... 15
 4.3 Primary endpoint ... 16
 4.4 Secondary endpoints ... 16
5. Trial design ... 16
 5.1 Summary of trial design ... 17
 5.2 Trial Schedule ... 19
6. Trial population ... 20
 6.1 Inclusion criteria ... 20
 6.2 Exclusion criteria ... 20
7. Recruitment, screening and informed consent ... 21
 7.1 Recruitment ... 21
 7.2 Screening ... 23
 7.3 Informed Consent ... 24
 7.4 Consent for long-term storage of biological material in a Research Biobank and a Biobank for future research ... 25
8. Randomization and blinding ... 25
 8.1 Randomization ... 25
 8.2 Blinding ... 25
9. Trial Interventions ... 26
 9.1 Structured supervised exercise training intervention ... 26
 9.2 Motivational counseling intervention supported by health technology ... 27
10. Investigation of endpoints ... 28
 10.1 Visit Schedule ... 28
 10.2 Procedures for obtaining measurements of primary and secondary endpoints ... 30
 10.3 Laboratory Tests ... 35
11. Assessment of safety ... 39
 11.1 Reporting ... 40
12. Data handling / Data Management ... 40
13. Sample size and statistical evaluation ... 41
 13.1 Sample size ... 41

13.2 Statistical analyses 42
13.3 Interim analysis 43
14. End of trial 43
14.1 Early termination of the trial 43
14.2 Participants lost to follow-up or discontinued from interventions 43
15. Administrative procedures 44
15.1 Organization 44
15.2 Facilities and infrastructures 44
15.3 Financing 45
15.4 Insurance 45
16. Publication 45
17. References 46

List of appendices

Appendix 1: Principal investigator agreement on clinical trial protocol
Appendix 2: List of investigators

List of Abbreviations

EXE	Structured supervised exercise training
MOT	Motivational counselling supported by health technology
CON	Control group receiving standard treatment
RCT	Randomized controlled trial
GA	Gestational age
PPAQ	Pregnancy Physical Activity Questionnaire
SF-36	The Medical Outcomes Study Short Form 36
PSQI	Pittsburgh Sleep Quality Index
P-ESES	Pregnancy Exercise Self-Efficacy Scale
BREQ-2	Behavioral Regulation in Exercise Questionnaire
DLW	Doubly labeled water
NOH	Nordsjælland's Hospital
UCPH	University of Copenhagen
WHO	World Health Organization
e-CRF	Electronic Case Report Form
AE	Adverse Event
SAE	Serious Adverse Event
PBMC's	Peripheral blood mononuclear cells
BMI	Body mass index
ASQ-3	Ages and Stages Questionnaire 3
PPD	Post Partum Depression
PSG	Polysomnography

1. Protocol Summary

**Title of trial:**	FitMum randomized controlled trial
**Trial ID:**	FitMum RCT
**Objectives:**	***The primary objective*** is to evaluate the effects of structured supervised exercise training (EXE) and motivational counseling supported by health technology (MOT) on physical activity level during pregnancy.<br>***The secondary objectives*** are ***1)*** to investigate the effects of EXE and MOT on complimentary measures of physical activity level, ***2)*** to qualitatively explore personal understandings of physical activity in everyday life of pregnant women and social, structural and cultural practices and factors influencing successful implementation of exercise training during pregnancy, ***3)*** to explore the way in which the FitMum RCT exercise programs are carried out and adapted, by conducting a process evaluation, ***4)*** to evaluate the effects of physical activity on clinical, and metabolic health parameters in mother and child, ***5)*** to evaluate the effects of maternal physical activity on growth and psycho-motor development in the offspring, ***6)*** to investigate the impact of pre-and postnatal maternal physical activity on offspring physical activity for 7 days at the age of 12 months, ***7)*** to evaluate the effects of physical activity during pregnancy on post partum mental well-being in mothers and fathers, and ***8)*** to explore the validity of activity trackers for measuring sleep among pregnant women.
**Trial design:**	FitMum RCT is designed as a single-site three-arm randomized controlled trial (RCT) and scheduled for startup on September, 2018. We will include 220 pregnant women as early in pregnancy as possible and no later than gestational age week 15+0 days (GA week 15+0). Included women will be randomly assigned to one of the three arms in the trial including a structured supervised exercise training (EXE) intervention, a motivational counseling intervention supported by health technology (MOT), and a control group receiving standard treatment (CON). The participants in the EXE and MOT groups continue in the physical activity intervention as long as their pregnancy allows and ideally until delivery, in total approximately six months. Collection of data for assessment of the primary endpoint will take place from randomization (one week after inclusion) to GA week 28+0-6 and will be measured by a commercial activity tracker. No interim analyses will be performed on the primary endpoint but may be performed on some of the secondary endpoints.
**Trial population:**	A total of 220 pregnant women will be included in the trial. Including 220 women in the trial will leave 70 women for statistical analysis in each of the two intervention groups and 35 in the control group, based on an expected dropout rate of 20% as seen in similar exercise studies in pregnant women. We will include physically inactive pregnant women aged 18 years or older with ultrasonic confirmed living intrauterine pregnancy before GA week 15+0 and whose health status allows them to exercise at moderate intensity.<br>12 months after delivery, offspring of participants will be invited to participate in a 7-day non-invasive tracking of physical activity level.

Methods: The effects of EXE and MOT on physical activity level will be measured by a commercial activity tracker, the Pregnancy Physical Activity Questionnaire and the doubly labeled water technique. Personal understandings of physical activity in everyday life of pregnant women and social, structural and cultural practices and factors influencing successful implementation of exercise during pregnancy will be investigated by qualitative interviews, observations and auto-documentation. Process evaluation of FitMum RCT interventions will be based on quantitative and qualitative data, e.g. qualitative interviews. Clinical and metabolic health parameters will be assessed by DXA scans, maternal and paternal blood samples, umbilical cord blood samples, placental samples, and questionnaires. Psycho-motor development in children will be assessed by questionnaire (ASQ-3) at 12 months of life, and physical activity will be monitored with a wrist-worn accelerometer for 7 days at 12 months of life. Parental mental well-being will be assessed by a validated screening questionnaire 6-8 weeks post partum. The validation of activity trackers for measuring sleep will be conducted using polysomnography (PSG) in a sub-group of forty women already participating in FitMum RCT.

Trial endpoints: ***The primary endpoint*** of the trial is physical activity level from randomization to GA week 28+0-6 determined by a commercial activity tracker.

Secondary endpoints include ***1)*** complementary measures of physical activity level, ***2)*** personal understandings of physical activity in everyday life of pregnant women and social, structural and cultural practices and factors influencing successful implementation of exercise during pregnancy, ***3)*** a qualitative and quantitative process evaluation of FitMum RCT interventions, ***4)*** clinical and metabolic health parameters in mother and child, ***5)*** growth, psycho-motor development and activity in the offspring, ***6)*** offspring physical activity at the age of 12 months, and ***7)*** post partum mental well-being in mothers and fathers.

Trial interventions: Included participants will be randomized in a 2:2:1 ratio to EXE, MOT or CON. The EXE intervention contains three weekly one-hour exercise sessions at moderate intensity, more specifically one water exercise session and two land exercise sessions. The training will be supervised, held in teams, and both water and land exercise sessions will consist of a combination of aerobic and resistance training. The MOT intervention contains four individual and three group counseling sessions taking place from randomization until GA week 33+6 and aim to motivate the participants to increase their physical activity level at moderate intensity. During individual sessions, feedback on physical activity performance will be provided based on activity data acquired from the activity tracker and further, MOT-participants will receive weekly SMS-reminders about physical activity.

Trial schedule:

Planned first participant first visit	3. quarter 2018
Planned last participant randomized	3. quarter 2020
Planned last participant last visit	2. quarter 2022
End of trial	3. quarter 2024

2. Flowchart

Visit number<br><br><br><br><br>Gestational age (week)	Visit 1<br>Screening + baseline testing<br><br>Max. 15+0	Telephone call<br>Randomi-zation<br><br>1 week after inclusion	Visit 2<br><br><br><br><br>Week 28+0-6	Visit 3<br><br><br><br><br>Week 34+0-6	Visit 4<br>Delivery<br><br><br><br>Approxima-tely week 40	Visit 5<br><br><br><br>7-14 days after delivery	1 year after delivery
Ultrasound scan	x						
Verbal information about trial	x						
Medical interview to assess in- and exclusion criteria	x						
Demographic and anthropometric data	x						
Record medical history, concomitant disease and previous medication	x						
Record medical history, physical activity level, demographic and anthropometric data of the father of the child	x						
Written Informed Consent	x						
Activity tracker and associated oral and written information is given	x						
Randomization		x					
ENDPOINTS							
Activity tracker		Continuously during the trial and 1 year after delivery					
Doubly labeled water			x				
Questionnaires: PPAQ, SF-36, PSQI, P-ESES, BREQ-2 and sickness absence + pelvic and low back pain	x		x	x			x
Sleep quantity and quality by the activity tracker		Continuously					
Maternal blood samples	x		x	x	x		
Paternal blood sample					x		
Umbilical cord blood samples					x		
Placental samples					x		
DXA scan						x	
Breast milk sample						x	
Sleep validation (PSG)			x				
Qualitative interviews		Continuously					
Questionnaires about physical activity in everyday life		Continuously					
Observation and auto documentation		Continuously					
ASQ-3 12-months post partum							x
Growth assessment and feeding-habits at 5 weeks, 5 months and 12 months							x

Parental mental well-being questionnaire, 6-8 weeks post partum.							x
7-day child accelerometer including anthropometry (clincal visit)							x
SAFETY							
Record Adverse Events			x	x			
Maternal weight measurement	x		x	x		x	x
Symphysis-fundal height measurement			x	x			

Table 1: Flowchart of measurements in FitMum RCT at visit 1-5 at Department of Gynecology and Obstetrics, Nordsjælland's Hospital (NOH). PPAQ: Pregnancy Physical Activity Questionnaire, SF-36: The Medical Outcomes Study Short Form 36, PSQI: Pittsburgh Sleep Quality Index, P-ESES: Pregnancy Exercise Self-Efficacy Scale, BREQ-2: Behavioral Regulation In Exercise Questionnaire, ASQ-3: Ages and Stages Questionnaire 3, PSG: polysomnography.

3. Background / Rationale

3.1 Trial rationale

Physical inactivity as a public health challenge in pregnant women

Low levels of physical activity during pregnancy constitute a significant public health issue as increasing evidence suggests that the mother's lifestyle during pregnancy may influence the health of her child (1,2). A physically active lifestyle during pregnancy shows potential to improve metabolic health of the child (3,4) and thus may play an important role in relation to counteracting the obesity epidemic and the increasing incidence of metabolic diseases that escalates globally (2,5).

The Danish Health Authorities recommend that healthy pregnant women are physically active for at least 30 minutes a day at moderate intensity (6), but fewer than four out of ten Danish pregnant women succeed to achieve the recommended level (7). Low physical activity level during pregnancy is a worldwide problem (8–12). Approximately 2/3 of the European WHO countries do not yet have national recommendations on physical activity during pregnancy (5) and the means to implement and maintain physical activity in everyday life are lacking (5). As the Danish annual birth rate has been around 60,000 children in the past years, and exactly 61,397 in 2017 (13), increasing physical activity among the current 60% of Danish pregnant women who are not achieving the recommended physical activity level will improve the health of over 35,000 Danish women and their children every year. As described in the following section about health benefits of physical activity during pregnancy for mother and child, previous research has reported positive effects of exercise for pregnant women's health including e.g. a reduction in gestational weight gain (14–20), intensity of low back pain (21) and risk of gestational diabetes mellitus (22–27). This can be expected to reduce sickness absence during pregnancy, reduce complications during delivery and a faster recovery of the mother after delivery. Moreover, it has been documented that physical activity during pregnancy is beneficial for the child by reducing e.g. the risk of preterm delivery (28,29) and normalizing birth weight (16). Finally, a healthier lifestyle during pregnancy might also impact health of mother and child after delivery, reducing the risk of obesity and cardiometabolic diseases. We hypothesize that the exercise programs will lead to significant economic benefits to the society in terms of higher productivity of mothers and reduced health care costs for both mothers and children. Thus, impacting more than 180,000 women and their children in only five years will have a significant economic impact.

We will assess the economic costs and benefits of the exercise programs and thus provide evidence on the cost-utility of FitMum RCT. The interventions are designed so the economic costs of the two exercise

programs are identical; the two exercise programs involve the same expenses and staff time per woman (more specifically 9 hours per woman for the entire intervention participation). Thus, costs will not make one of the regimes more attractive than the other. In order to assess the economic benefits of the interventions, we will collect information about sickness absence during pregnancy from the program participants. We will build on state-of-the-art health economic methods combined with the high-quality Danish data on health care costs (Diagnosed Related Grouping data and other types of register based cost data) to assess cost savings for the healthcare sector as a result of improved health for mothers and children. Data collection for the economic evaluation will be conducted as an integral part of the collection of health data on intervention and control groups.

In Denmark, only very few and scattered regional or municipal physical activity initiatives are targeted pregnant women, and these are mainly targeted overweight pregnant women (30). The absence of concrete recommendations on how to implement a physically active lifestyle during pregnancy and the lack of public initiatives also represents a key contributor to social inequality in health among pregnant women, as mainly women with both social and economic resources may prioritize to participate in one of the physical activity initiatives offered in the private sector.

The longer lasting vision of the FitMum RCT trial is to fill the massive gap in evidence and practice and provide the public sector with the evidence of the effects of two different exercise programs that can be made available to all pregnant women, independent of the social economic position. Thereby, FitMum RCT has the potential to form the basis for development of new public initiatives that meet the pregnant women’s needs for flexible and non-costly exercise training and matches their individual wishes and preferences towards physical activity.

The mother’s and child’s health benefits of physical activity during pregnancy

Increasing physical activity levels among pregnant women is crucial, as regular physical activity during pregnancy promotes several clinical and metabolic health benefits in mother and child, and it reduces the number of complications during pregnancy and delivery (3,31–33). A growing body of evidence of the positive health effects of physical activity during pregnancy exists with findings originating from observational studies, cross-sectional studies, high-quality randomized controlled trials and systematic reviews and meta-analyses of randomized controlled trials.

In terms of the child’s health, regular physical activity during pregnancy has been shown to normalize birth weight (16), reduce risk of preterm delivery (28,29), improve neonatal body composition (34,35) as well as placental function (36,37), resulting in improved intrauterine growth conditions. In a recent cohort study, higher levels of physical activity during pregnancy was associated with longer duration of breastfeeding (38). Moreover, animal studies have shown that physical activity during pregnancy improves insulin sensitivity and glucose tolerance in the offspring (39–41). The underlying mechanisms by which the lifestyle of the mother influences the later health of the child are however sparsely known. Several reviews (1,2,4) have suggested that fetal programming, via epigenetic changes in the fetus, may constitute the underlying mechanisms, and point out the need for clinical studies in humans to investigate the extend and the mechanisms through which maternal exercise training improves metabolic health in the next generation. Physical activity during pregnancy has in animal models improved learning and memory in of the offspring. Only very few human studies have addressed the psycho-motor development of children in respect to maternal activity level during pregnancy. Two studies identified a positive impact of physical activity during pregnancy on language development in the offspring (42,43) and a recent review including 6 studies concluded that physical activity during pregnancy was positively associated with total neurodevelopment and specific language neurodevelopment in the first 18 months of life (44). Physical activity in the first years of life have positive effects on multiple health outcomes (45). Maternal level of physical activity has been associated with physical

activity in 9-month Chinese infants (46). However, no study has investigated a possible programming effect of maternal physical activity during pregnancy assessed by an accelerometer on offspring physical activity.

Regular physical activity during pregnancy also affects the health of the mother by, among others, reducing gestational weight gain (14–20), risk of gestational diabetes mellitus (22–27), intensity of low back pain (21), caesarean delivery (16,24,47–50) and improving maternal body composition (51). Metabolic benefits of physical activity during pregnancy appear to include improvement of glucose tolerance (23,26,52), plasma lipid profile (53), and concentrations of insulin (54,55), leptin (55), interleukin-6 (54) and C-reactive protein (56).

Regular physical activity during pregnancy may also affect mental well-being in parents after delivery. Postpartum depression (PPD) is a prevalent illness, and a recent Danish study found that 8% of fathers and 11% of mothers showed significant signs of PPD (57), supporting other studies that have found PPD in 4% - >60% of women in the first year after giving birth (58–60). PPD negatively affects parents functioning, personal relationships, and offspring's social and cognitive development (61). Several systematic reviews and meta-analyses found evidence that exercise may be effective in reducing depressive symptoms during pregnancy (62) and the postpartum period (63–65). A recent review, although based on a small number of studies, suggests that physical activity during pregnancy may likewise serve to prevent subsequent PPD, particularly in previously inactive women (66).

Pregnancy as a window of opportunities and limitations

Although health effects of physical activity are widely acknowledged, the means to implement and maintain physical activity in everyday life are lacking (5). Pregnancy can be regarded as a window of opportunity to implement good habits of physical activity, as the women are in regular contact with health professionals and may be motivated to adopt healthy behaviors as evidenced by reduced alcohol consumption and smoking cessation (2,5,67). For others, pregnancy can be conceived as an opportunity to be exempt from demands about fitness and bodily ideals, or be experienced as a difficult and troublesome time with fatigue and discomfort (68,69). Moreover, pregnancy is a short period of time when it comes to changing habits (69). This can affect the motivations and challenges for physical activity for each participant. Furthermore, differences in work status, social relations, family situation and material and structural conditions may contribute to make implementation of physical activity a complex task (Bønnelycke, personal communication).

To explore important motivational factors and barriers to physical activity during pregnancy, we recently carried out 27 semi-structured qualitative interviews with Danish pregnant women, midwives and obstetricians. We included pregnant women with different ages, physical activity levels, gestational age, educational levels, civil status, number of children, socioeconomic status, geographical location of residence, and pre-pregnancy body mass index (BMI), as motivators and barriers to physical activity have been suggested to be associated with pre-pregnancy BMI (70). Results from the interviews have been exploratively, inductively and thematically analyzed under the supervision of Associate Professor Astrid Jespersen, SAXO-Institute, University of Copenhagen (UCPH). The findings from the interviews, yet unpublished data, indicated the following key attention points to be included in the design of the FitMum RCT interventions:

- In terms of motivators, the interviewed pregnant women pointed to pregnancy as a window of opportunity to implement healthier lifestyles. They further highlighted advice and feedback from experts, and introduction to types of physical activity that are feasible to implement as important motivators to adapt an active lifestyle during pregnancy.

- On the other hand, the interviewed women pointed out anxiety of over-doing exercise and confusing and contradicting advices from relatives and healthcare professionals as key barriers to be physically active.

The women also pointed out a shifting energy level throughout the pregnancy and lack of time as barriers to be physically active.

These barriers to physical activity during pregnancy are supported by international literature (71) concluding that interventions to encourage the women to meet the recommended levels of physical activity in pregnancy should be accompanied by accessible and consistent information about the positive effects for mother and child, and that midwives should be encouraged to promote physical activity in pregnancy (71). However, a recent survey from the UK concluded that only two percent of midwives could correctly identify the physical activity guidelines during pregnancy, with 25% giving incorrect responses and 29% being unsure of what the guidelines were (72). Thus, pregnant women may experience inconsistent advice and recommendations.

Furthermore, pregnant women are longing for personalized advice and recommendations instead of receiving only the general advice on physical activity that has been designed for all pregnant women (73). The latest recommendations on lifestyle interventions during pregnancy support the idea of favoring individualized advice about how to increase the physical activity level rather than applying a one-size-fits-all approach (69). It is important to investigate different physical activity approaches as women may have different preferences regarding how, when and where to be physically active. Some women prefer to participate in a supervised exercise training program at specific times during the week and in groups, while others prefer to go for a walk or go bicycling at self-chosen times (15 and own preliminary results from semi-structured qualitative interviews).

Taken together, among policy makers, healthcare professionals and pregnant women, there is an enormous need and request for consistent evidence-based guidelines on how to implement physical activity in everyday life during pregnancy in a safe and effective manner, and for approaches that meet the needs, preferences and barriers of the pregnant women. This will increase the confidence of being physically active among pregnant women and encourage and empower pregnant women to increase their physical activity levels.

The FitMum RCT approaches to implement physical activity during pregnancy

Structured supervised exercise training and motivational counseling constitute two very different approaches to implement and maintain a physically active lifestyle. The majority of physical activity intervention studies in pregnant women are conducted on overweight and obese populations (17,18,20,23,74–81). Fewer studies have investigated healthy normal-weight pregnant women (14,15,21,27,82–85) and none of these have focused primarily on investigating the effect of their exercise intervention on actual physical activity levels in pregnant women, or used objective methods to measure physical activity levels. This contributes strongly to the massive gap in evidence of the effects of physical activity interventions and how to implement physical activity programs in healthy pregnant women’s everyday life. Both of the approaches examined in FitMum RCT have been applied separately in pregnant women (14,15,17,18,20,21,23,27,76–87), but the relative efficacy of structured supervised exercise training and motivational counseling has not previously been compared in pregnant women, which hampers evidence-based implementation of effective exercise programs in daily practice.

As described below, the objective of the FitMum RCT trial is to generate evidence about **how** to implement physical activity in healthy pregnant women’s everyday life. We will test the effects of the above-mentioned two very different exercise approaches versus a control group on primarily physical activity level, which will be measured objectively by commercial activity trackers in all three trial groups. The two exercise programs are based on structured supervised exercise training and motivational counseling, respectively. The interventions have been designed to meet the motivators and overcome the specific barriers to physical activity among pregnant women based on insights from our qualitative interviews and available national and international

recommendations for physical activity during pregnancy (3,6,88,89), and available literature within the field (69,86).

Structured, supervised exercise training constitutes a classic and widely used approach to implement physical activity, and as mentioned above, this approach has also been applied previously with pregnant women as target group (14,15,21,23,27,77,78,82,90,91). Applying structured supervised exercise training will most likely accommodate several of the above-mentioned findings from our qualitative interviews regarding motivators and barriers to implement a physically active lifestyle during pregnancy. For example, the highlighted motivations “advice and feedback from experts” and “introduction to types of physical activity that are feasible to implement” will be available within a structured supervised exercise training intervention. Furthermore, the highlighted barrier “anxiety of over-doing exercise” might be accommodated and prevented by the close monitoring of training sessions from trained instructors. The content of the training sessions can be adjusted appropriately to the individual woman ad hoc during training sessions in close collaboration between the woman and the exercise instructors. However, the barrier “lack of time” that were also found in the analysis of our qualitative interviews, is considered to be a challenge for pregnant women as the structured supervised exercise training approach has training sessions that are held at fixed time points. In FitMum RCT, we aim to overcome this barrier related to fitting physical activity into the everyday life of pregnant women by offering training sessions at six to eight different times per week, including weekday mornings and afternoons, as well as weekends.

Motivational counseling constitutes another approach to increase physical activity level and this approach has also been applied in both pregnant (17–20,26,92) and non-pregnant (93–98) populations. This exercise approach will most likely accommodate the same motivators for implementing a physically active lifestyle, as described for the structured supervised exercise training approach, since motivational individual and group counseling sessions are also assumed to be able to provide “advice and feedback from experts” and “introduction to types of physical activity that are feasible to implement”. The barrier “anxiety of overdoing exercise” as highlighted in the qualitative interviews is also assumed to be prevented by applying this exercise approach. In addition, motivational counseling is characterized by encouraging implementation of physical activity in a more flexible manner, which is in line with the focus of a new Danish political initiative from January 2018 targeting future efforts within the birth area in order to promote a good and safe start in life (99). A flexible approach to implementation of physical activity seems promising in order to prevent the barrier concerning “lack of time”, since pregnant women may find it easier to fit physical activity into their everyday life by applying this approach. However, by attending motivational counseling to increase physical activity, pregnant women still need to allocate some time for being physically active, which can be challenging. In FitMum RCT, we aim to overcome this barrier by trying to schedule the individual counseling sessions in accordance with the women’s regular control visits at Nordsjælland’s Hospital (NOH).

Several theoretical frameworks exist for this exercise approach and in FitMum RCT we will apply motivational interviewing and self-determination theory (100) and behavior change techniques (101). In addition, besides using the commercial activity trackers to objectively measure physical activity levels in all three FitMum RCT trial groups, the trackers will be used as an intervention element motivating to increase the physical activity level among the pregnant women in the motivational counseling group, hereby supporting our motivational counseling intervention by health technology. We plan to support our motivational counselling intervention by health technology as it has been shown that including an activity tracker in behavioral physical activity interventions can lead to an increased physical activity level (94,102) and that new health technologies constitute a major opportunity for implementing, motivating and maintaining physical activity in everyday life (103,104). Commercial activity trackers have been heavily developed as part of the digital revolution and are more and more frequently used for research purposes (105–107). Therapeutic application of self-monitoring of physical activity via activity trackers carries a great potential (107), and in addition, continuous assessment of

physical activity provides an opportunity to markedly advance our understanding of the impact of frequency, duration, quality and intensity of physical activity on human health and well-being.

As mentioned earlier, the relative efficacy of structured supervised exercise training and motivational counseling has not previously been compared with pregnant women as target group. However, the effects of approaches that are somewhat similar to structured supervised exercise training and motivational counseling have been compared in another population: A study conducted by one of our international collaborators, Ralph Maddison, and his colleagues, compared the costs and outcomes associated with community support (mimicking structured supervised exercise training) and motivational telephone counselling, in 60-year old New Zealanders (108). Community support included weekly face-to-face support group meetings in which physical activity was offered, whereas phone support involved monthly telephone calls over a 3-4-month period, whereby patients were assisted with goal setting, action planning, provided with information and were encouraged to participate in physical activity. The findings suggested similar costs and effects on health outcomes with the two exercise approaches, but a higher level of physical activity in the community support group (108). This favors the structured supervised exercise training approach if the aim is to increase physical activity level. However, physical activity was self-reported in the study by Maddison and colleagues and therefore associated with an inherent bias, and thus these results should be carefully interpreted. On the other hand, intervention studies conducted in pregnant women have found a positive effect on physical activity level by applying interventions containing motivational individual and group sessions that encourage to increase the physical activity level (17,18,79).

Thus, the previous results regarding the effects of structured supervised exercise training compared to motivational counseling are conflicting and a direct comparison on the effects of these two approaches on physical activity level in pregnant women is needed in order to generate evidence about **how** to implement physical activity in healthy pregnant women’s everyday life.

3.2 Benefit-risk assessments and ethical considerations

The FitMum RCT will adhere to the principles of the Helsinki declaration. Before initiation, the trial protocol has been approved by the Danish Data Protection Agency (Datatilsynet, # VD-2018-336) and registered at the clinical database, www.clinicaltrials.gov (# NCT03679130).

Regular physical activity at moderate intensity is recommended for pregnant women (3,6,88,89). Physical activity during pregnancy is widely acknowledged to improve health of mother and child as well as reduce the number of complications during pregnancy and delivery (3,31–33). By participating in FitMum RCT, the individual participant is given the opportunity to engage in an exercise intervention free of charge and increase her physical activity level during pregnancy. In addition, she will be provided with relevant health information about herself and her child.

Despite the fact that pregnancy is associated with profound anatomic and physiological changes, exercise has minimal risks in pregnant women and their fetus (3,16,24,29,84,109–112). The risks associated with participation in the trial are considered minimal, since both interventions are developed based on current recommendations on physical activity for pregnant women (3,6,88,89).
The pregnant women will be informed in the written participant information that they might experience increased heart rate, fatigue and muscle- and pelvic soreness when being or after being physically active, which is, however, considered harmless.

Wearing a wrist band accelerometer (Actigraph GT3X+) for 7 days at 12 months of life is not expected to carry a risk for the child. Previous studies in infants has proven wearable accelerometers to be a safe, feasible and reliable way to monitor physical activity (46,113,114).

It can be perceived as a disadvantage that participants must spend time filling out questionnaires electronically, and theoretically some parents may become unnecessarily worried. Participants will have easy access to sparring with the research team if there is concern about their child's well-being and development. Similarly, the extra screening for signs of postpartum depression provides extra assurance that parents will receive the necessary help and support if needed.

4. Objectives and endpoints

4.1 Primary objective and hypotheses

The primary objective of FitMum RCT is to evaluate the effects of structured supervised exercise training (EXE) and motivational counseling supported by health technology (MOT) on physical activity level during pregnancy.

Our hypotheses are that both EXE and MOT will increase the physical activity level of the pregnant women and that MOT will increase the activity level to the same extent as EXE.

4.2 Secondary objectives

1. To investigate the effects of EXE and MOT on complimentary measures of physical activity level

2. To qualitatively explore personal understandings of physical activity in everyday life of pregnant women and social, structural and cultural practices and factors influencing successful implementation of exercise training during pregnancy with the following aims:

- To explore family relations, health practices, self-perception and experience of pregnancy, as well as practical and environmental/structural constraints such as family dynamics, logistics and time
- To explore how the two exercise programs appeal to women from different socioeconomic groups
- To investigate whether the costs and efforts associated with the interventions (e.g. sports clothing and shoes, fitness membership fees, transport to/from gym/swim bath, alongside the practical managements of family and work life) are perceived as prohibitive for exercising

3. To explore the way in which the FitMum RCT exercise programs are carried out and adapted by conducting a process evaluation with the following aims:

- To explore if the FitMum RCT exercise programs were delivered as intended (e.g. fidelity and dose)
- To understand whether, how and why the programs have an impact, through exploring providers' and participants' perspectives of the interventions (and the standard treatment)
- To explore implementation barriers and facilitators of a complex intervention
- To explore if the results are likely to be generalizable, scalable and sustainable through exploring stakeholders' (hospital, municipalities, providers, participants) experiences of the programs and their perceived impact (health economics)

4. To evaluate the effects of physical activity on clinical and metabolic health parameters in mother and child, in order to investigate underlying mechanisms in relation to metabolic and epigenetic adaptations to physical activity during pregnancy.

5. To evaluate the effects of maternal physical activity on growth, psycho-motor development and activity in the offspring

6. To investigate the impact of pre-and postnatal maternal physical activity on offspring physical activity at the age of 12 months

7. To evaluate the effects of physical activity during pregnancy on post partum mental well-being in mothers and fathers

8. To explore the validity of activity trackers for measuring sleep among pregnant women.

4.3 Primary endpoint

The primary endpoint of FitMum RCT is physical activity level from randomization to GA week 28+0-6 determined by a commercial, wrist-worn activity tracker with built-in heart rate monitor and accelerometer.

4.4 Secondary endpoints

The secondary endpoints are divided into seven categories and include:

1) Complimentary measures of physical activity level
2) Data within a qualitative project investigating personal understandings of physical activity in everyday life of pregnant women and social, structural and cultural practices and factors influencing successful implementation of exercise during pregnancy
3) Qualitative and quantitative process evaluation of FitMum RCT interventions
4) Clinical and metabolic health parameters in mother and child

5) Growth and psycho-motor development in the offspring

6) Pattern and level of daily activity in the offspring at 12 months of age

7) Post partum mental well-being in mothers and fathers

Please refer to section 10 for a detailed description of primary and secondary endpoints including procedures for obtainment of measurements and analyses of endpoints.

5. Trial design

5.1 Summary of trial design

FitMum RCT is designed as a single-site three-arm randomized controlled trial (RCT). The trial is scheduled for startup on September 2018, and is expected to continue until the end of 2022. The practical aspects of the trial are to be carried out at Department of Gynecology and Obstetrics at NOH by the investigators, primarily two PhD-students Signe de Place Knudsen and Caroline Borup Roland, a postdoc and master students affiliated with FitMum RCT. A total of 220 pregnant women will be included as early in pregnancy as possible and no later than GA week 15+0. During the trial the interventions (the three arms) will only be applied to the pregnant women. To gain a broader understanding on the effects of exercise during pregnancy on the child's health, we will obtain data from the father of the child (blood sample and demographic data) at visit 1 and from the child (umbilical cord blood, placental samples, clinical data on neonatal outcomes, e.g. birth weight) at visit 4. Children's growth, psycho-motor development and activity will be followed during the first year of life, and parental mental well-being will be assessed 6-8 weeks after delivery. Pattern and level of physical activity in the offspring will be assessed at 12 months of age using a wrist worn accelerometer for 7 days (Actigraph GT3X+).

After inclusion, baseline physical activity level will be measured for one week by a commercial activity tracker. Hereafter, each participant will be randomly assigned to one of the three arms in the trial including a structured supervised exercise training (EXE) intervention, a motivational counseling intervention supported by health technology (MOT), and a control group receiving standard treatment (CON). The EXE and MOT interventions will start immediately after randomization and pregnant women in these groups continue in the physical activity interventions as long as their pregnancy allows and ideally until delivery, in total approximately six months. Physical activity level, which is the primary endpoint, will be assessed by the commercial activity tracker from randomization to GA week 28+0-6. During the time period of the trial, the participants visit NOH five times for measurement of secondary endpoints as described below. The trial design including interventions elements and measurements is illustrated below in Figure 1. No interim analysis will be performed on the primary endpoint but may be performed on some of the secondary endpoints.

Figure 1 - Trial design

Timeline	EXE	MOT	CON
GA week<br>6-10	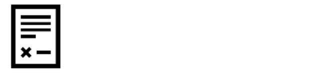	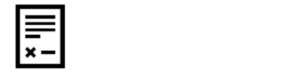	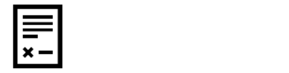
Max. GA week 15+0<br>**(Visit 1: part 1 - screening)**	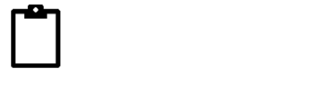	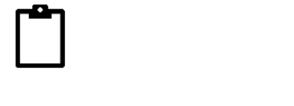	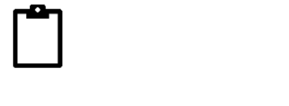
Max. GA week 15+0<br>**(Visit 1: part 2 – baseline tests)**	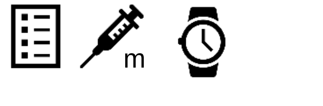	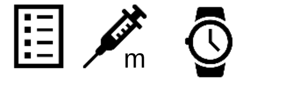	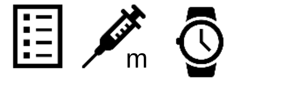
1 week after Visit 1	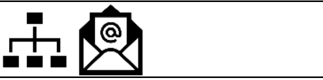	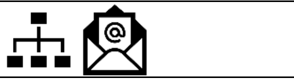	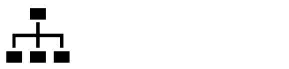
Randomization -<br>3 weeks after randomization	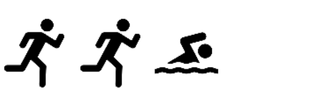	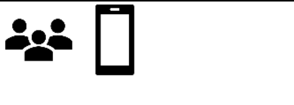	
4–6 weeks after randomization	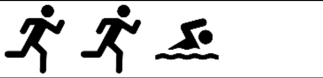	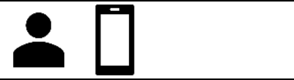	
Between individual session 1 and group session 2	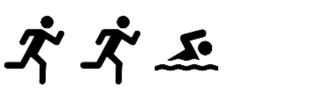	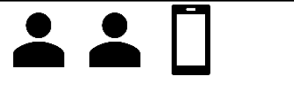	
GA week<br>24+0 – 26+6	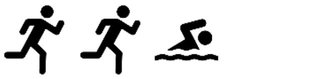	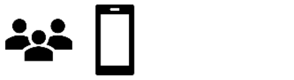	
GA week<br>27+0-6	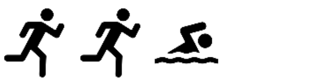	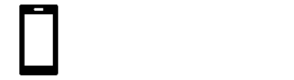	
GA week<br>28+0-6<br>**(Visit 2)**	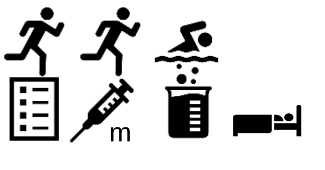	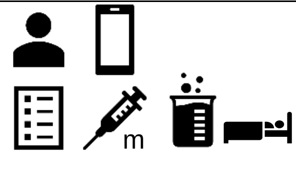	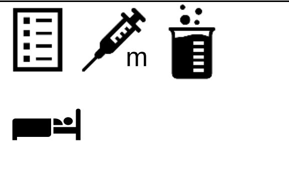
GA week<br>29+0 – 30+6	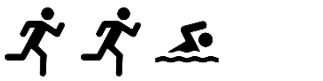	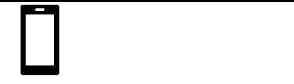	
GA week 31+0 – 32+6	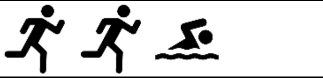	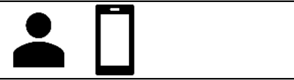	
GA week 33+0 – 33+6	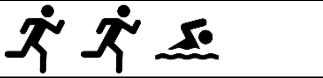	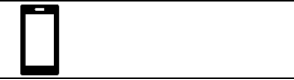	
GA week<br>34+0-6<br>**(Visit 3)**	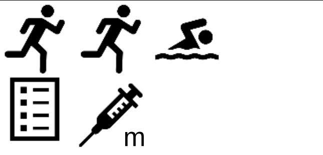	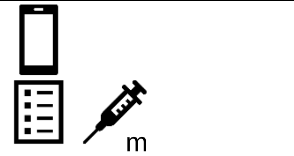	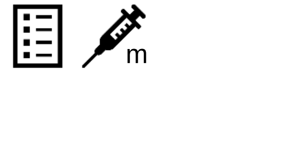
GA week<br>35+0 – 37+6	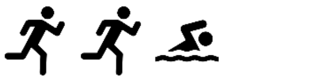	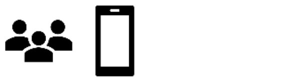	
GA week<br>38+0 – 39+6	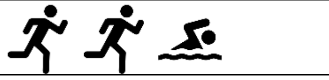	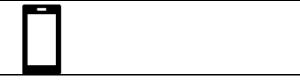	

CONFIDENTIAL

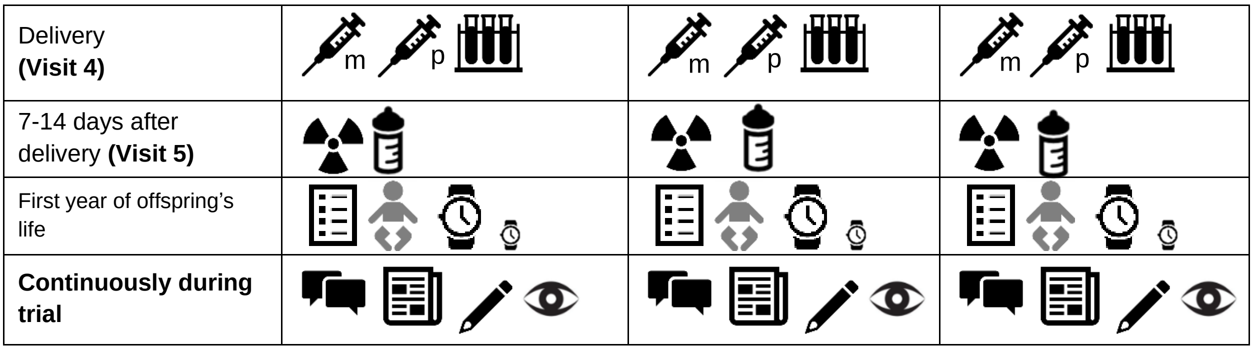

Figure 1. FitMum RCT intervention elements and measurements at visits at NOH are illustrated by pregnancy weeks in all three groups. EXE: Structured supervised exercise training group, MOT: Motivational counselling supported by health technology group, CON: Control group. PPAQ: Pregnancy Physical Activity Questionnaire, SF-36: The Medical Outcomes Study Short Form 36, PSQI: Pittsburgh Sleep Quality Index, P-ESES: Pregnancy Exercise Self-Efficacy Scale, BREQ-2: Behavioral Regulation In Exercise Questionnaire, ASQ-3: Ages and Stages Questionnaire 3, PSG: polysomnography.

Written information about the trial
Verbal information, medical interview and written informed consent
Questionnaires: PPAQ, SF-36, PSQI, BREQ-2, P-ESES and sickness absence + pelvic and low back pain in pregnancy, ASQ-3 and parental mental well-being postpartum
m Maternal blood samples
p Paternal blood samples
Attachment of activity tracker
Interviews
Observation
Randomization
Electronic flyer about physical activity
Weekly land exercise session
Weekly water exercise session
Group counseling session
Individual counseling session
Auto-documentation
Weekly SMS-reminder
Questionnaires about physical activity in everyday life
Drinking doubly labeled water
Placenta and umbilical cord blood samples
DXA scan
Breast milk sample
Growth and development
7-day 24h infant activity tracker 12 months after delivery
PSG

5.2 Trial Schedule

Planned first participant first visit	3. quarter 2018
Planned last participant randomized	3. quarter 2020
Planned last participant last visit	2. quarter 2022
End of trial	3. quarter 2024

6. Trial population

We will include 220 physically inactive pregnant women before GA week 15+0 whose health status allows them to exercise at moderate intensity. Women meeting all of the inclusion criteria listed and none of the exclusion criteria will be considered eligible for the trial.

6.1 Inclusion criteria

1. Written informed consent obtained before any trial related procedures are performed
2. Pregnant woman aged 18 years or older
3. Gestational age of max. 15+0 weeks
4. Ultrasonic confirmed intrauterine pregnancy
5. BMI of 18.5-45 kg/m^2 or a weight below 150 kg calculated from pre-pregnancy weight or first measured weight in pregnancy

6.2 Exclusion criteria

1. Severe chronic disease
2. Structured exercise at moderate to vigorous intensity more than 1 hour per week during pregnancy
3. Previous preterm delivery (before GA week 37)
4. Obstetric or medical complications
5. Multiple pregnancies
6. Non-Danish speaking
7. Alcohol or drug abuse

7. Recruitment, screening and informed consent

7.1 Recruitment

Recruitment will occur through three channels. All three channels provide the same information about the trial to potential participants (Figure 2) but will be presented via different environments and platforms. We apply this broad recruitment strategy by several different channels in order to attempt to minimize recruitment bias.

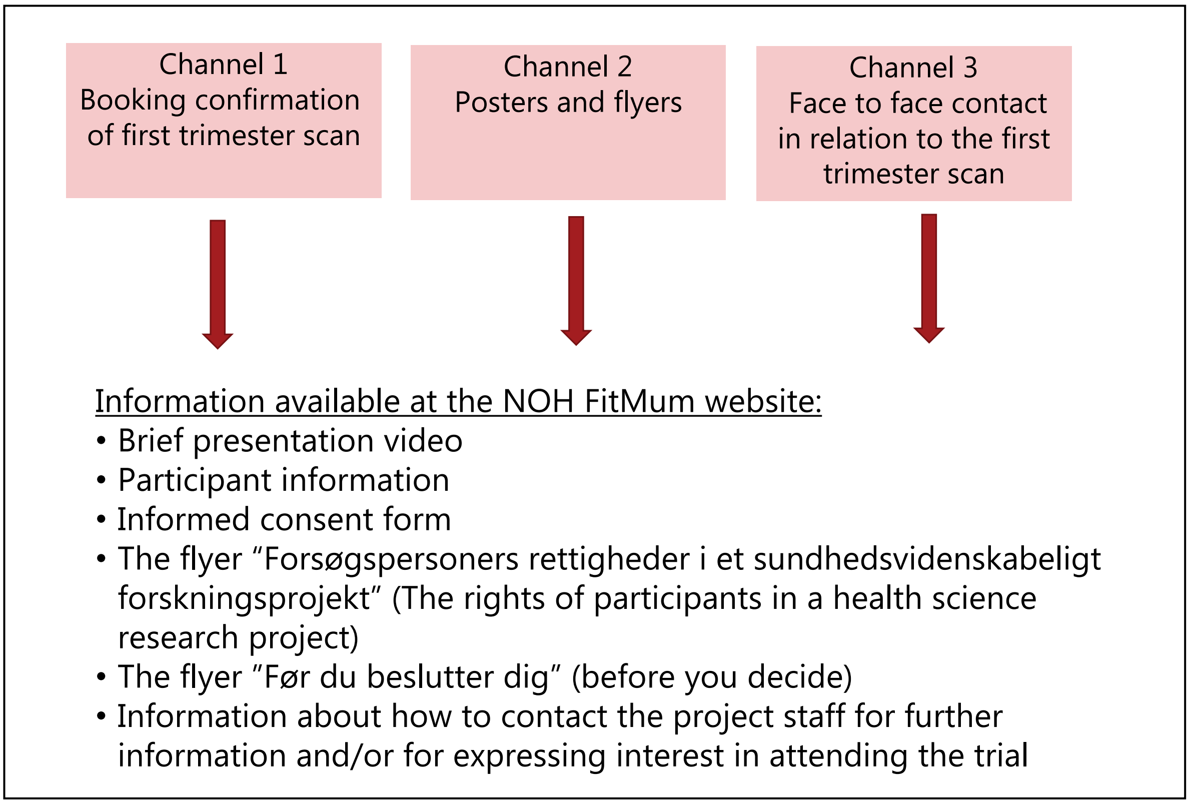

Figure 2: Overview of recruitment channels and information made available to potential participants in FitMum RCT.

<u>Channel 1 – Booking confirmation of the first trimester scan:</u>

All women registered for delivery at NOH will book their first trimester scan via NOH's website, if they are interested in attending the scan. When having booked the scan, the women receive a confirmation note on the computer screen. Within this confirmation note there will be brief information about the trial including a link, from which the women can be directed to the NOH website presenting the information shown in Figure 2. At this website, there will be a link, from which the women can be directed to the prescreening questionnaire, which they must fill out, if they are interested in participating in the trial. Furthermore, the women will receive the first trimester scan confirmation via email, which will also include the above mentioned brief information and link to the NOH website.
Almost all women registered for delivery at NOH accept the offer of attending a first trimester scan and book the scan online. Therefore, most pregnant women registered for delivery at NOH will receive information about FitMum RCT through this channel. Hence, the channel is important in order to attempt to minimize recruitment bias.

Channel 2 – Posters and flyers:

Posters and flyers will be exposed at a) NOH's website, b) social medias, c) in waiting rooms at local general practitioners, at gynecological specialist doctors, at an ultrasound clinic in Hillerød, and at pharmacies and health shops etc. in the recruitment area, d) at the Department of Gynecology and Obstetrics at NOH. The posters and flyers will contain the same information about FitMum RCT as the brief presentation video (https://region-hovedstaden-ekstern.23video.com/fitmum-wmv-1) that will be available on the NOH website (Figure 2).

a) As mentioned in channel 1, all women registered for delivery at NOH must independently book their first trimester scan via NOH's website, if interested in attending the scan. At the same website, a small FitMum RCT info box will be visible including a link, from which the women can be directed to the NOH website presenting the information shown in Figure 2.

b) A poster with information about the trial will be exposed at social medias including local Facebook pregnancy groups, www.min-mave.dk etc. Posts on websites will include a link, from which the women can be directed to the NOH website presenting the information shown in Figure 2.

c) Pregnant women visit their general practitioner in GA weeks 6-10. In the waiting rooms at local general practitioners there will be posters hanging and flyers to bring home. Posters and flyers will also be available at gynecological specialist doctors in the region, at an ultrasound clinic in Hillerød, and at pharmacies and health shops etc. in the recruitment area. Interested women can use the QR-code at the posters and flyers, which will be linked to the NOH website presenting the information shown in Figure 2. If the women cannot use the QR-code access, they can contact the PhD-students directly for request of more information by email.

d) Some pregnant women visit the Department of Gynecology and Obstetrics at NOH before the first trimester scan. At the acute and elective Departments, there will be posters hanging and flyers to bring home. Interested women can use the QR-code at the posters and flyers, which will be linked to the NOH website presenting the information shown in Figure 2. If the women cannot use the QR-code access, they can contact the PhD-students directly for request of more information by email.

Channel 3 – Face to face contact in relation to the first trimester scan:

All women registered for delivery at NOH will be offered a first trimester scan at GA week 11+2-14+1. The scan takes place at the Department of Gynecology and Obstetrics at NOH. While in the waiting room, the women will be contacted face-to-face by the PhD-students. The women will be informed briefly about the trial and if interested, she will be asked a few questions (from the prescreening questionnaire described below), to prescreen whether she is eligible for inclusion. If the woman meets the relevant criteria and are interested in being further screened for inclusion in the trial, she will on paper or email depending on her preference, receive the participant information, informed consent and the two flyers "Forsøgspersoners rettigheder i et sundhedsvidenskabeligt forskningsprojekt" and "Før du beslutter dig" (Figure 2). Further, the PhD-students will obtain the woman's contact details and written informed consent to receive the prescreening questionnaire by email (if not answered at NOH) and be contacted by the PhD-students within the following two days if she has not contacted the PhD-students by herself. If interested after having read the documents, an appointment at NOH, where screening for inclusion in the trial will take place (visit 1), is scheduled with the PhD-students.

7.2 Screening

Screening for inclusion in the trial will occur in two steps including a prescreening questionnaire and a screening visit at NOH (visit 1, Figure 1).

Prescreening questionnaire:

The woman must respond to a one-page questionnaire about her physical activity level in the first trimester of the pregnancy, whether she can speak and understand Danish, and if she previously has given birth prematurely (before GA week 37). If recruited through channel one or two, the questionnaire will be administered electronically by email where the woman receives a link to the questionnaire that is administered via the electronic database, REDCap, which is further described in section 12. If recruited through channel three, the woman can either answer the questionnaire in the waiting room at Department of Gynecology and Obstetrics at NOH or choose to receive an email with a link to the questionnaire and answer it electronically as applied for women recruited through channel one and two. The link directing interested women to the prescreening questionnaire can also be found on the NOH website presenting information about the trial.

Visit 1:

If the woman appears to be eligible for inclusion based on the prescreening questionnaire she is invited to visit 1 at the Department of Gynecology and Obstetrics at NOH as soon as possible and no later than GA week 15+0 (see Figure 1). Visit 1 includes two elements: ***a screening session*** and subsequent ***baseline testing*** if the woman is included in FitMum RCT.

Women entering the screening via recruitment channel 1 or 2 (before the first trimester scan) must be ultrasound scanned (to confirm a living intrauterine pregnancy) immediately after obtainment of informed consent to participate in FitMum RCT. Women entering the screening via recruitment channel 3 will be scanned at the first trimester scan. Since the first trimester scan is a part of the already existing pregnancy and delivery program at NOH this ultrasound scan is not considered a specific FitMum RCT trial procedure (as if the ultrasound scan is performed before the first trimester scan), and can therefore be done before obtainment of informed consent for participation in the trial.

The screening session includes an ultrasound scan to confirm a living intrauterine pregnancy and a medical interview. The interview takes place in an undisturbed room and the woman has been informed prior to the visit that she may bring her partner, a family member or a friend. This may aid the decision-making, of whether or not to join the trial. At the interview, the woman will be verbally informed about the trial and screened according to the in- and exclusion criteria in order to decide, whether she can be included in the trial. If she can be included, demographic and anthropometric data and background information will be obtained. Demographic data include date of birth, educational level, job status, civil status, number of children, and smoking status. Anthropometric data include height, pre-pregnancy weight and current weight. The same data will be obtained from the father of the child if he is the biological father. If the woman is interested in participating in FitMum RCT, written informed consent is obtained from her as described in section 7.3.

Besides obtaining written informed consent from the pregnant woman, written informed consent is also obtained from other holders of custody (described in section 7.3), since FitMum RCT includes obtainment of biological samples from the child and the father of the child (if biological father). Biological samples obtained from the child includes obtainment of umbilical cord blood samples and placental samples at delivery, and the biological sample obtained from the father includes a paternal blood sample that will be obtained at visit 4. We encourage both the mother and the other holder of custody to participate in visit 1, so that both can be verbally informed

about their participation in FitMum RCT at this visit. Written informed consent about the child's participation will be obtained from both parents if shared custody at visit 1 or right after. Further, written informed consent about obtainment of paternal blood sample will be obtained from the father of the child (if biological).

If written informed consent is obtained directly after the interview at visit 1, the woman will proceed directly to baseline testing. Further, the paternal blood sample will be obtained immediately hereafter. Otherwise, the baseline tests will be scheduled as soon as possible after visit 1. If it is not possible for both holders of custody to attend visit 1, the holder of custody not present will be offered a new appointment for an interview with the PhD students at NOH in order to receive verbal information about their child's participation in the trial and obtainment of the paternal blood sample (if biological father). If written obtained consent is obtained from the father (if biological) directly after this interview the paternal blood sample will obtained immediately hereafter. Otherwise, a new appointment for obtaining written informed consent and the paternal blood sample will be scheduled as soon as possible after this interview.

After signing the informed consent (no later than GA week 15+0), the woman will undergo the ***baseline tests*** including obtainment of blood sample and answering five questionnaires (see Table 1). Further, she will receive a commercial activity tracker, which has to be worn on the wrist 24/7 for a one-week baseline test and then throughout the trial. During the trial, compliance with wearing the activity tracker and syncing of data will be continuously monitored by the members of the research team through a research platform associated to the trackers.

7.3 Informed Consent

As FitMum RCT includes data from primarily the pregnant woman, but also from the child, and the father of the child (if biological father), written informed consent is being obtained from both the woman herself and other holders of custody.

The pregnant woman:

The written informed consent is being obtained from the pregnant woman before any trial activities are performed.

Through recruitment channel 1-3 and visit 1, respectively, all women will be in writing and verbally informed about the trial. The woman will be informed that participation in the trial is voluntary and that she may withdraw from the trial at any time during the course of the trial and that withdrawal of consent will not affect her subsequent pregnancy and delivery process at NOH. The woman will be given time to ask questions and allowed 24 hours to deliberate on trial participation before obtainment of written informed consent.
It is the responsibility of the PhD students to obtain the written informed consent from the woman.

After completion of trial, all participants will be informed of the overall results. Information of individual data and the interpretation hereof will be provided by the investigators. The individual participant's right not to know of own data will be respected.

Custodial parents:
Prior to visit 1, holders of custody will receive written information regarding umbilical cord blood samples, placental samples at delivery, a paternal blood sample at visit 4, questionnaire on parental well-being at week 6-8 post partum and monitoring of offspring's growth, psycho-motor development and activity. We encourage both holders of custody to participate in visit 1, where holders of custody will be verbally informed about their child's and the father's (if biological) participation in FitMum RCT. Holders of custody are informed that their child's participation in the trial is voluntary and that they may withdraw their child from the trial at any time during the

course of the trial and that withdrawal of consent will not affect their or their child's subsequent process at NOH. The same information is given to the father of the child (if biological) regarding obtainment of a blood sample from him at visit 4. Again, holders of custody will be given the opportunity to ask questions and are allowed 24 hours to consider the trial before deciding whether to participate.

7.4 Consent for long-term storage of biological material in a Research Biobank and a Biobank for future research

When the women and other holders of custody are asked to consent to the participation in the trial, they will also be asked specifically if they can accept the storage of their biological material (maternal and paternal blood, umbilical cord blood, placental and urine samples) in coded form within a Research Biobank and later storage of excessive biological material (maternal and paternal blood, umbilical cord blood, placental and urine samples) in a Biobank for future research. The biological material in the Research Biobank will be analyzed continually and probably within 5 years after (01.06.2025) the last participant has completed the trial (01.06.2022). Samples will be stored at - 80^0 C. The purpose of storing samples in the Research Biobank is to analyze all samples at the same time in order to minimize data variation. After analyses are performed, excessive biological material (maternal and paternal blood, umbilical cord blood, placental and urine samples) will be transferred to the biobank for future research at NOH and stored at – 80^0 C, so that subsequent analysis of additional markers can be performed without further discomfort to the participant. Samples in the Biobank for future research will be stored for 15 years (01.06.2040) provided participants' acceptance; hereafter the biological material in the Biobank for future research (maternal and paternal blood, umbilical cord blood, placental and urine samples) will be destroyed according to the guidelines of Nordsjælland's Hospital. Additional analysis will only be performed after approval by the Ethical Committee System on Biomedical Research Ethics and by the Danish Data Protection Agency (Datatilsynet). Further, the participant will be contacted in order to obtain new informed consent for carrying out the analyses, unless the Ethical Committee System assesses that this is not necessary. Participants are able to participate in the trial without giving consent to long-term storage of their biological material in the Biobank for future research.

8. Randomization and blinding

8.1 Randomization

After the one-week baseline period where physical activity level is measured by the activity trackers, participants are randomized in a 2:2:1 ratio to EXE, MOT or CON. The target number of women randomized to each group is 88, 88 and 44, respectively. Randomization will be performed using computer software to create a numbered randomization list. The randomization list will be generated by a trial-independent colleague at NOH and will not be accessible to investigators involved in the conduct of the trial, until the database has been locked. After identification of an eligible participant, the participant will be assigned a randomization number. The investigators must always allocate the lowest available randomization number to the participant. The randomization number is a four-digit number starting at 1001 and with this all participants enrolled will be identifiable throughout the trial. Participants will be informed about which group they are randomized to by a telephone call. Pregnant women in the EXE and MOT groups will start in the exercise interventions immediately after randomization.

8.2 Blinding

The investigators at FitMum RCT will be blinded to the randomization procedure as described in section 8.1. Data collected for assessment of primary and secondary endpoints will be statistically analyzed by the PhD

students and blinded by the principal investigator. Blinding of participants is not attempted as it is considered impossible due to the inherent content of the exercise interventions.

9. Trial Interventions

Each participant will be randomly assigned to receive a structured supervised exercise training (EXE) intervention, motivational counseling supported by health technology (MOT) intervention or standard treatment (CON). Participants randomized to the CON group will be included in the already existing pregnancy and delivery program at NOH. They will be informed that it is important to continue to live as they would have done without participation in FitMum RCT, which is especially important in relation to physical activity.

The target physical activity level for participants in EXE and MOT is at least 30 minutes per day at moderate intensity as recommended for healthy pregnant women (6). Both exercise interventions will start immediately after randomization after the one-week baseline period. After randomization, participants in the EXE and MOT groups receive an electronic information flyer by email containing guidelines about physical activity during pregnancy from the Danish Health Authorities, information about the benefits of physical activity during pregnancy, examples of safe and unsafe physical activities during pregnancy, tips for increasing physical activity levels, e.g. ‘use the stairs instead of the elevator’, ‘use your bike for transportation to work’, and links to small videos with exercises or to websites providing information of local opportunities for being physically active. FitMum RCT intervention elements and measurements are illustrated in Figure 1.

9.1 Structured supervised exercise training intervention

The structured supervised exercise training (EXE) intervention will be delivered face-to-face* by two PhD students (Caroline Borup Roland and Signe de Place Knudsen) and master students. The intervention consists of three weekly one-hour exercise sessions at moderate intensity including two land exercise sessions and one water exercise session. In particular, water exercise* has been identified as a beneficial, popular and safe activity for pregnant women (7,115,116). The training will be supervised, held in teams, and both water and land exercise sessions consist of a combination of aerobic and resistance training. The participants are instructed to exercise at moderate intensity during all training sessions, which is accommodated by asking the participants to appropriately increase the number of repetitions and resistance of exercises corresponding to exercising at moderate intensity. The training sessions include relative progression over time as the participants are expected to improve their technical skills and fitness during the intervention period. Moderate intensity during training sessions will be assessed using both heart rate of 65-80% of age-predicted maximal heart rate (from the activity tracker) and rating perceived exertion in the range of 12-14 on Borg’s conventional 6-20-point scale (117), as recommended by the American College of Obstetricians and Gynecologists (3). Further, content of a training session will be adjusted to the individual participant ad hoc during the session, e.g. one participant taking fewer repetitions in/not doing e.g. a back-exercise if she experiences back pain on that specific day.
A private Facebook group established for all participants in the EXE intervention group will be used as a communication platform between participants and FitMum RCT staff to remind the participants to attend training sessions, sharing pictures from training sessions, encouraging the participants to post music wishes for the training playlists etc.

*COVID-19 in Spring 2020 has forced us to change the way we deliver the EXE intervention. The water sessions are cancelled until the facilities open again. All sessions are land exercises, 30 minutes of aerobic exercise where the women exercise on their own, followed by 30 minutes of supervised group resistance training on Zoom Cloud Meetings. We will return to the original intervention when it is allowed.

9.2 Motivational counseling intervention supported by health technology

The motivational counseling intervention supported by health technology (MOT) will be delivered face-to-face** and by SMS-reminders by Caroline Borup Roland, Signe de Place Knudsen and master students. The intervention consists of four individual and three group counseling sessions with a coach as well as weekly SMS-reminders encouraging a moderate physical activity level. Further, a social network is established in terms of a private Facebook group for all participants in the MOT group, in order to provide the opportunity to communicate and e.g. self-organize common physical activities with other pregnant women from the local area.

Individual and group counselling sessions are scheduled like this:

Group session 1:	max 3 weeks after randomization
Individual session 1:	4-6 weeks after randomization
Individual session 2 and 3:	distributed evenly based on the times of individual session 1 and group session 2
Group session 2:	GA 24+0 – 26+6
Individual session 4:	GA 31+0 – 32+6
Group session 3:	GA 35+0 – 37+6

**COVID-19 in Spring 2020 has forced us to change the way we deliver the MOT intervention. All individual and group sessions have been online using Zoom Cloud Meetings.

Individual counseling sessions:

All individual sessions will last one hour and the overall focus is on motivating the pregnant woman to increase her physical activity level. Positive and negative experiences and emotions towards physical activity will be elicited and acknowledged and the participant's perspectives of advantages and disadvantages about changing towards a more physically active lifestyle will be discussed. In cooperation, the counselor and the participant map the participant's personal wishes, needs and former physical activity experiences to identify individual characteristics and motivation. The participant sets/resets her own individual goal(s) of physical activity and identifies where and how the participant can secure social support regarding physical activity engagement. It is of great importance that the participant herself makes choices regarding her physical activity wants and needs. An individual action plan for increasing physical activity level will be made in collaboration between participant and counselor. The provided amount of information about physical activity will vary between participants dependent on the individual needs and interests. During all individual sessions, feedback on recent physical activity performances will be provided based on activity data acquired from the activity tracker. The participant's physical activity data will be provided in order to give the participant the opportunity of following and measuring, to what extent she reaches her own personal goal for physical activity, which can have a motivating effect on physical activity behavior (94,102). Further, the data will be used to appropriately adjust the individual goal setting and action planning to fit in to the participant's current everyday life and state of pregnancy, continually aiming for the overall target of achieving at least 30 minutes of daily physical activity at moderate intensity. The focus will vary a bit from session to session, e.g. being on how to be physically active in third trimester and after birth in the last individual session in GA week 31+0 – 32+6.

Group counseling sessions:

Two to five participants, who are more or less at the same stage in their pregnancies, and a counselor, will participate in the group sessions. The first group session lasts one hour and aims to inform the participants about guidelines for physical activity, benefits associated with being physically active during pregnancy, and possible ways of being physically active during pregnancy. A cooperative and emphatic relationship between the participants is aimed to be build and the counselor will be a leading facilitator and guide the participants through this session. In the following two two-hour group sessions, the interaction between the participants is

used to create meaningful group processes such as support, experience exchange, reflection, learning and development. These sessions will focus on discussion of relevant topics in relation to physical activity during pregnancy and the counselor will be a facilitator through the session, but the topics of conversation will be chosen by the participants and their needs and wants, based on previously set up wishes and expectations for this session by every participant. Counseling sessions (individual and group) are designed with a progression in autonomy over time meaning that the content of the first group and individual counseling session will be led by the counselor and gradually, in respect of every individual participant, the participants can help determine the content of the sessions. Some of the individual- and group sessions will be taped for quality and consistency assurance. The PhD students will continually review tapes and discuss best practice.

Weekly SMS-reminders:

Finally, another element of the MOT intervention is that participants are offered weekly SMS-reminders, which content will be supportive and motivating in relation to increase physical activity level.

10. Investigation of endpoints

10.1 Visit Schedule

The primary endpoint is measured continuously during the trial, and moreover the participants visit the Department of Gynecology and Obstetrics at NOH five times for measurement of secondary endpoints during the trial period. The procedures performed for investigation of endpoints at all visits at NOH are illustrated in Table 2 below and all measurements obtained at the visits are presented in Table 1 in section 2. The procedures presented in Table 2 are listed in the chronological order in which they should preferably be performed:

Visit ID	Procedures to be performed
**Visit 1 *<br>(Screening and baseline testing)<br><br>Max. GA week 15+0**	*Screening:*<br>Perform ultrasound scan to confirm a living intrauterine pregnancy<br>Provide verbal information about the trial<br>Medical interview to assess compliance with inclusion and exclusion criteria<br>Record medical history, concomitant disease and previous medication<br>Obtain demographic and anthropometric data<br>Obtain medical history and physical activity level from the father of the child<br>Obtain written informed consent for the trial<br><br>*Baseline testing if included in the trial:*<br>Obtain maternal blood samples<br>Provide activity tracker and associated information<br>Questionnaires are electronically administered (PPAQ, SF-36, PSQI, P-ESES and BREQ-2) and information about sickness absence + pelvic and low back pain is obtained.
**Telephone call<br>(Randomization)<br><br>1 week after visit 1<br>Max. GA week 16+0**	Randomize the participant<br>Instruct the participant about content of assigned group<br>Participants in the EXE and MOT groups receive a flyer by email about physical activity during pregnancy

Visit ID	Procedures to be performed
**Visit 2 *** <br>**GA week 28+0-6**	Obtain maternal blood samples<br>Administer DLW and provide instructions about urine samples collection<br>Questionnaires are electronically administered (PPAQ, SF-36, PSQI, P-ESES and BREQ-2) and information about sickness absence + pelvic and low back pain is obtained.<br>Record AEs and concomitant medication<br>Measure maternal weight<br>Obtain symphysis-fundal height measurement<br>PSG sleep study in a subgroup of participants
**Visit 3 *** <br>**GA week 34+0-6**	Obtain maternal blood samples<br>Questionnaires are electronically administered (PPAQ, SF-36, PSQI, P-ESES and BREQ-2) and information about sickness absence + pelvic and low back pain is obtained.<br>Record AEs and concomitant medication<br>Measure maternal weight<br>Obtain symphysis-fundal height measurement
**Visit 4 *** <br>**Delivery**	Record AEs and concomitant medication<br>Obtain maternal and paternal blood samples<br>Obtain umbilical cord blood samples within 30 min. after delivery<br>Obtain placental samples within 30 min. after delivery
**Visit 5 *** <br>**7-14 days after delivery**	Record AEs and concomitant medication<br>Perform DXA scan of mother<br>Obtain breast milk samples<br>Measure maternal weight
**First year of offspring's life**	Questionnaires are electronically administered (SF-36, PPAQ, PSQI, P-ESES, BREQ-2 and ASQ-3 12 months post partum, and Parental mental well-being 6-8 weeks post partum)<br>The women measure their own weight<br>Obtain data on growth and feeding habits from parents (5 weeks, 5 months, 12 months post partum)<br>Data on activity tracker from mothers (365 days)<br>7-day 24h infant activity tracker at 12 months of life

Table 2: Procedures performed at the five visits at Department of Gynecology and Obstetrics, NOH. EXE: Structured supervised exercise training group, MOT: Motivational counselling supported by health technology group, GA week: Gestational age week, PPAQ: Pregnancy Physical Activity Questionnaire, SF-36: The Medical Outcomes Study Short Form 36, PSQI: Pittsburgh Sleep Quality Index, P-ESES: Pregnancy Exercise Self-Efficacy Scale, BREQ-2: Behavioral Regulation In Exercise Questionnaire, ASQ-3: Ages and Stages Questionnaire 3, DLW: doubly labeled water, PSG: polysomnography

* COVID-19 in Spring 2020 has forced us to change the procedures in FitMum temporarely, until we are allowed to return to normal practice. From March 11th all interventions and visits have been online, using Zoom Cloud Meetings (DEiC-secured via licences from UCPH) or telephone. When included the desinficted tracker and consent will be delivered outside the home of the women. If they don't have a weight, the will be weight at home on a weight brought to them by FitMum at the same visit. No blood samples are optained at the visits, the women are weighed at home, and symphysis-fundal height measurements are not measured. No double labled water is administered at visit 2. The womens weight at visit 4 are noted by the midwifes on the day of giving birth. No DXA-scans or breast milk samples are collected at visit 5.
From June 1st 2020 we will start taking samples at visit 4 again, since the hospital allows research sampling on patients who are admittet. We will return to the original protocol, interventions and samplings at all visits, when it is allowed.

10.2 Procedures for obtaining measurements of primary and secondary endpoints

This section provides a detailed description of the primary and secondary endpoints illustrated in Table 1 and outlines the trial procedures performed for obtaining measurements of endpoints. For further details on the specific timing of the procedures please refer to Table 2 in section 10.1.

Primary endpoint

The primary endpoint of FitMum RCT is physical activity level measured from randomization to GA week 28+0-6 determined by a commercial, wrist-worn activity tracker with built-in heart rate monitor and accelerometer (see Table 1). The activity tracker is to be worn continuously by the women from inclusion until delivery. Preferably, the activity tracker is also worn continuously from delivery and one year ahead. The activity tracker determines the frequency, duration and intensity of activity periods on a minute-to-minute basis and data from the activity tracker is wirelessly synced to its associated APP and research platform. One of the major advantages of measuring physical activity level by commercial activity trackers is that data are collected and transferred automatically to a smartphone or website, which makes continuous assessment of physical activity over months or even years possible (107).

The wrist-worn commercial activity trackers and oral instructions about the trackers are provided to the participants at visit 1. The participants also receive a flyer in paper and by email with practical information about the activity tracker. During the trial, compliance with wearing the activity tracker and syncing of data will be continuously monitored through a research platform associated to the trackers.

Secondary endpoints

- ***Complementary measures of physical activity level:***

In addition to determining physical activity level by the commercial activity tracker, physical activity will be measured by a Danish version of the validated ‘Pregnancy Physical Activity Questionnaire’ (PPAQ) and by the doubly labeled water technique (DLW). This ensures complimentary information on activity tracker measured physical activity level (118). Also, physical activity level of the mother in the first year after delivery will be determined by activity tracker. The participant will have to weight herself at home six times during the first year after delivery and change her weight registration in the Garmin Connect App. The woman will receive an email reminder about this.

The PPAQ is a semi-quantitative and subjective instrument that has been validated in pregnant women (119) and is identified to be among the most valid and reliable questionnaires for assessment of physical activity level in pregnant women (120). It is adapted from the International Physical Activity Questionnaire developed by the WHO and can distinguish between different types of activities, e.g. house hold chores, occupational activities and sports. PPAQ will be administered electronically by email at visit 1 (no later than GA week 15+0), 2, 3 and one year after delivery. It will be send with a deadline for answering of five days. If the questionnaire is not answered within five days, an SMS-reminder will be send with a deadline for answering of two days and this procedure is repeated one time if needed.

Doubly labeled water (DLW) is ‘gold standard’ technique for objectively measuring free-living energy expenditure and is safe, even for pregnant women, as it relies on stable, non-radioactive isotopes (121–124). Before administering DLW, two baseline urine samples are collected on two different days the week before Visit 2. After the baseline urine samples are collected, the participant is administered a glass of water for oral intake containing 0.1 g of $^{2}H_2O$ at 99.98 atom percent ^{2}H and 0.16 g of 100% ^{18}O per kg body weight. Following the

day of DLW administration, a total of five post-dose urine samples of at least 10 mL each are collected in the post-absorptive state in the morning; on the day after oral water dosage, on day four, seven, 11 and on day 14, respectively. The urine sample must not be the first urine void of the day. The reason for this is that the bladder acts as a reservoir for urine that is generated throughout the night reflecting an integrated sample over an extended time rather than a sample reflecting the urine concentration at a specific time point. The urine samples are stored in a freezer and delivered to the research staff as soon as possible (for further information, see section 10.3). DLW will be administered at visit 2 and urine samples will be returned at NOH two weeks after. A manual about how to collect and store the urine samples are given to the participants.

- ***Personal understandings of physical activity in everyday life of pregnant women:***

This secondary endpoint includes data within a qualitative project investigating personal understandings of physical activity in everyday life of pregnant women and social, structural and cultural practices and factors influencing successful implementation of exercise during pregnancy.

The methodology employed for data collection will mainly be qualitative and conducted in sub-groups consisting of approximately 10 participants from each of the three trial groups, aiming at including a range of participants with different socio-economic status (educational level, employment status, relationship status, etc.). Methods to be used are:

- Qualitative semi-structured interviews
- Observations of visits at NOH
- One to two whole days of participant observation on an intervention day (EXE or MOT)
- One week of auto-documentation repeated each trimester, beginning as close to the entry in the trial as possible
- Postpartum follow-up interviews, participant observation and auto-documentation (photos + diary for a week) six months and one year after the intervention

Short individual qualitative interviews with all women will be conducted at the beginning and end of the project period. Qualitative investigations will be scheduled in accordance with each participant’s personal calendar and will preferably take place outside the clinical setting, if possible at the participants’ homes. All women are requested to answer a questionnaire administered by email during the project period.

As non-compliance, non-participation and loss to follow-up could provide important indicators of challenges to implement physical activity, there will be focus on recruiting and interviewing women who discontinue from interventions for personal reasons or for any of the reasons mentioned in section 14.2. These women will be invited to an interview about the reasons for discontinuance, experience of participation, and of performing exercise training during pregnancy.

Additionally, it is reasonable to presume that those most at risk for excessive gestational weight gain and living inactive lifestyles are those least likely to participate in the interventions. Therefore, the qualitative studies will include a sub-group of socio-economically disadvantaged or vulnerable pregnant women, who do not take part in the interventions. These will be recruited via the NOH special efforts team (‘Tidlig Indsats’), focusing on pregnant women with social, physical or mental challenges. This will be a group that is particularly difficult to get in contact with, and this will therefore be approached with extra care and in close collaboration with the ‘Tidlig Indsats’ team and the midwives closest to the women. The aim is to include 10 participants, preferably more if possible. The methods will be adjusted to each participant’s situation and abilities, which are expected to vary

greatly. The methods will preferably be:

- Observations of visits at NOH and participation in the 'Tidlig Indsats' program
- Qualitative interview(s) with participants, preferably at the same GA week times as intervention groups
- One week of auto-documentation repeated each trimester, beginning as close to the entry in the trial as possible
- Postpartum follow-up interviews, and auto-documentation (photos + diary for a week)

The investigations will focus on the participants' physical and mental health and wellbeing, social relations and physical activity, and the experience of pregnancy in relation hereto.

This part of the trial is expected to provide insights into the practices, rationales and barriers of those who might arguably benefit most from being physically active, but due to the social, physical and mental challenges have the greatest impediments for participation. These insights can be of great importance to provide socially sensitive and contextually adjusted advice and support for physical activity during pregnancy.

- ***Process evaluation of FitMum RCT:***

FitMum RCT is a complex and interdisciplinary intervention trial containing several interacting components with varying degrees of difficulties of behaviors required by the provider and the receiver of the interventions (125). A study published in British Medical Journal shows that in only half of the studies published on non-pharmacological interventions in well accepted journals, leave information about which components that are included in the interventions (126). To legitimize the development of the interventions and place it in the context of other published and ongoing trials (127), FitMum RCT is designed and developed based on principles for complex interventions. The development phase has been conducted using a thorough literature study, available recommendations for physical activity during pregnancy (3,6,88,89), the latest recommendations on intervention design for pregnant women (69,86), and insights from a pilot study based on 27 semi-structured qualitative interviews with pregnant women, midwives and obstetricians.

A process evaluation will be conducted alongside the trial to explore the way in which the FitMum RCT exercise programs are carried out and adapted. A process evaluation is in line with the Medical Research Council guidance on evaluating complex interventions in health (125). If the FitMum RCT programs are found effective, a key question is how the FitMum RCT programs can be generalized, scalable and implemented outside a trial setting. A process evaluation can provide valuable insight into the mechanisms through which interventions bring about change, why an intervention leaves behind unexpected results, or how a successful intervention works and can be optimized. Common features in process evaluations of intervention-implementation are reach, dose, adherence, adaptions, fidelity, quality of delivery, participant responsiveness, and context. There is still no consensus on how best to define and divide these subcomponents. However, fidelity is generally understood as the extent to which an intervention has been implemented by the professionals as planned (125). The process evaluation will be used to assess the fidelity and quality of implementation, clarify causal mechanisms, and identify contextual factors associated with variation in outcomes (128,129). Key functions of process evaluation and relationships amongst them are illustrated in Figure 3.

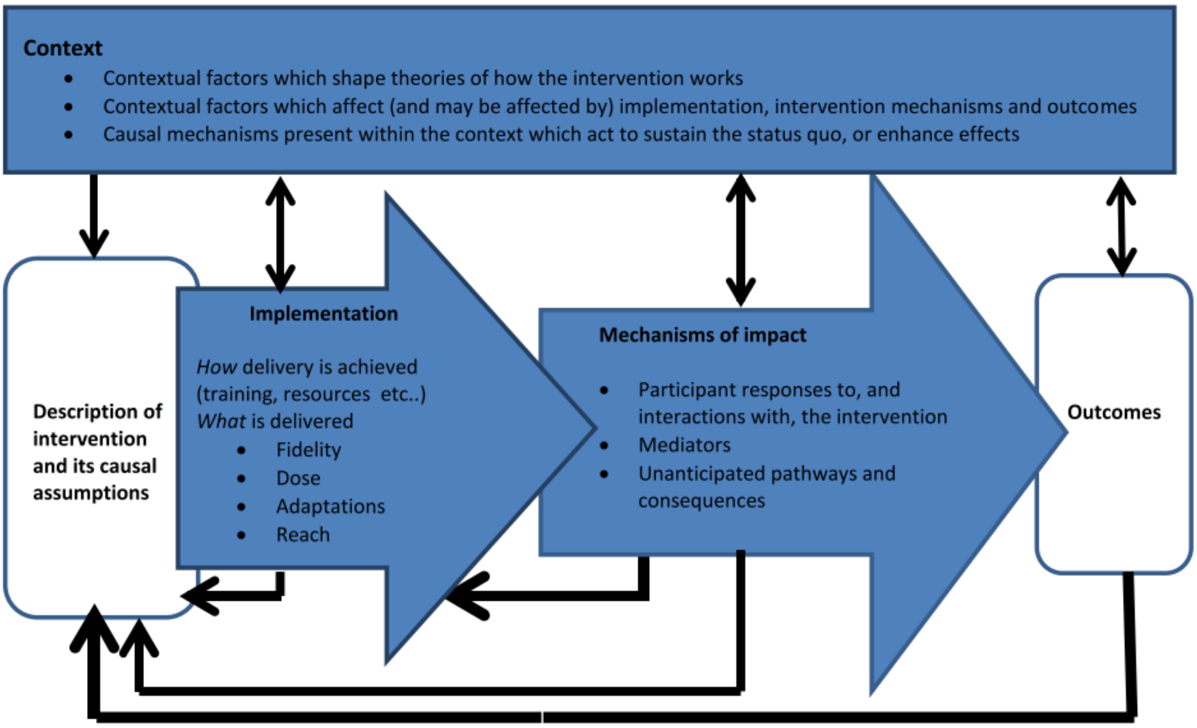

Figure 3: Key functions of process evaluation and relationships amongst them (blue boxes represent components of process evaluation, informed by the intervention description, which inform interpretation of outcomes (125).

In FitMum RCT, women in the EXE and MOT groups will receive a flyer containing guidelines about physical activity during pregnancy and information about the benefits of physical activity during pregnancy, which reflects an assumption that increased knowledge of physical activity may trigger a behavioral change (125). If no process evaluation is made, the assumption cannot be assessed. As stated within the Medical Research Council guidance for evaluating complex interventions, it is possible to "build a cumulative understanding of causal mechanisms, design more effective interventions and apply them appropriately across groups and settings" by understanding the implementation and functioning of interventions in a given context (130).

The process evaluation of FitMum RCT will apply a case study design focusing on different events within a given context. The context will be studied primarily from individual- and intervention-level perspectives rather than from a system-level perspective by use of both quantitative and qualitative data. The focus will be on the mechanisms that lead the interventions to work or not work and to open the "black box" between providing and receiving or developing and implementing (130–132). Data will be collected alongside the implementation of the two interventions. Quantitative data will be collected to assess the reach of the intervention, the dose and fidelity.

Through an analysis of the administratively collected process data (e.g. frequency of training and counselling sessions, visits and measurements), electronic case record files, and activity logs (e.g. measurements of the total time spent per woman in all three trial groups), it will be explored if FitMum RCT was delivered as intended.

The qualitative interviews will be used to understand processes, including the disclosure of knowledge, values, preferences and needs of participants and providers. Through semi-structured interviews conducted with participants and providers to understand their perspective of any causal mechanisms of the interventions and to seek their suggestions on how the exercise programs could be scaled up and rolled out. Findings related to

questions about participants' perspectives of what they expect post-trial in relation to the programs will also be explored. Participants for the interviews will be recruited using a large variation sampling (variables to be considered for the women: intervention group EXE, MOT and CON, physical activity level, age, parity etc. Variables to be considered for providers: educational level, seniority, weight of "hands on/hands off" etc.). The interviews will be individually held or held in teams. It is estimated, that 30 interviews will be held and analyzed.

- ***Clinical and metabolic health parameters in the mother, father and child:***

Clinical data about pregnancy, delivery and neonatal outcomes will primarily be collected from medical records at NOH after visit 4.

Maternal body composition during pregnancy will be assessed using DLW at visit 2 and by DXA scan at visit 5. The procedures regarding urine sampling applied for investigating body composition by DLW is the same as described above where DLW is used to estimate free-living energy expenditure and hereof, physical activity level. Applying the DLW method, maternal body composition during pregnancy will be determined from total body water determined from the post-absorptive $^{2}H_2O$ enrichments (133). During DXA scan a weak x-ray radiation is used. The scan lasts approximately 10 minutes and the radiation dose is approximately 3-30 µSv corresponding to less than 1 week of background radiation.

Mental well-being and health-related quality of life and function will be determined by

a) a Danish version of the Medical Outcomes Study Short Form 36 (SF-36) (134,135) that has been validated also in pregnancy (136). It will be administered at visit 1, 2, 3 and one year after delivery, and by the same procedure as for PPAQ as described above.
b) a Danish validated screening-tool for parental post-partum depression, developed by Rigshospitalet (57) primarily based on the Edinburgh Postnatal Depression Scale (137,138) and selected questions from The Masculine Depression Scale (139). It will be administered to the mother and father 6-8 weeks post partum.

Sickness absence and pelvic and low back pain will be assessed by obtaining information from the participant at visit 1, 2 and 3.

Sleep quantity and quality will be assessed continuously by the activity trackers and by the Pittsburgh Sleep Quality Index (PSQI) (140) at visit 1, 2, 3 and one year after delivery. PSQI is a self-administered questionnaire that measures sleep quality over a period of 1-month (141). PSQI is considered a valid and reliable tool to assess sleep metrics among pregnant women (142). This questionnaire will be administered by the same procedure as for PPAQ and SF-36 as described above.

Validation of activity tracker for measuring sleep: A one-night validation study of the activity tracker for measuring sleep will be performed in a subgroup of FitMum participants. The "gold standard" method for measuring sleep is polysomnography (PSG). The ambulatory PSG consists of a device (the size is half a smartphone) and a number of electrodes that can be attached to the head. The electrodes and the device itself can annoy the participants and limits movement, but otherwise has no side effects (143). Forty participants are recruited among pregnant women already participating in the FitMum study. The participant will during the day have the ambulatory PSG set up with instructions on how to turn it on and off. The participants will at home turn the device on at bedtime and sleep, as usual, that night; the study ends when the participant wakes up in the morning and takes off the device.

Exercise self-efficacy will be determined by a Danish version of Pregnancy Exercise Self-efficacy Scale (P-ESES)(144). It will be administered at visit 1, 2, 3 and one year after delivery by the same procedure as for PPAQ as described above.

Physical activity motivation will be determined by a Danish version of The Behavioural Regulation In Exercise Questionnaire (BREQ-2) (145–147) which is the most widely used measures of the continuum of behavioural

regulation in exercise psychology research. It will be administered at visit 1, 2, 3 and one year after delivery by the same procedure as for PPAQ as described above.

Plasma metabolites, hormones and DNA methylation of maternal and paternal peripheral blood mononuclear cells (PBMC's) will be assessed by obtaining maternal blood samples at visit 1, 2, 3 and 4 and a paternal blood sample from the father of the child (if biological father) at visit 4. Blood samples will be taken using standard venipuncture techniques. A total of 20 mL will be collected per participant per visit. In total, maximally 80 mL blood per pregnant woman will be obtained during the trial. A total of 20 mL will be collected from the father at visit 4. Obtainment of blood samples can cause transient soreness and a minor hematoma.

Plasma metabolites, hormones and DNA methylation of umbilical cord blood cells are assessed by obtaining arterial and venous blood samples from the umbilical cord at visit 4 within 30 minutes after delivery of the placenta. A total of approximately 10 mL umbilical cord blood will be collected. This causes no additional burden for mother or child, and under normal circumstances the umbilical cord will be discarded after delivery.

Placental function will be assessed from placental samples (from both decidua and chorion side) taken at visit 4 within 30 minutes after delivery of the placenta. The samples will immediately be frozen in liquid nitrogen and stored at -80 ^{0}C. This causes no additional burden for mother or child, and under normal circumstances the placenta will also be discarded after delivery.

Exercise-induced adaptations to the breast milk will be assessed from one breast milk sample obtained by the participant at Visit 5. The sampled amount is approximately 2.5 mL and will be taken anytime during the day and from one single feed. The sample should contain a "full feed" to account for any variation between fore- and hind milk. The samples will be stored at -80 ^{0}C.

Growth and feeding habits of the offspring 0-12 months. Body weight, body length and head circumference are assessed at 5 weeks, 5 months and 12 months as part of the national routine preventive childhood examinations. Parents will be asked to reply a brief questionnaire on their childs growthand feeding habits at 5 weeks, 5 months and 12 months.

Psycho-motor development of the offspring will be assessed by the validated "Ages and Stages Questionnaire 3". This questionnaire pinpoints developmental progress in the fields of communication, gross motor, fine motor, problem solving, and personal-social skills. When the ASQ is used as a screening test, better-than-average children score near maximum, resulting in an underestimation of the mean. To overcome this statistical problem, and to get a normal distribution of the ASQ raw scores, other studies have evaluated children with questionnaires meant for older children (148). In the current study the ASQ-questionnaire for children of 12 and 14 months of age will be administered electronically to parents when the child is 12 months of age.

Physical activity of the offspring. At age 12 months the pattern and level of physical activity in the offspring will be assessed by a 7-day 24h infant activity tracker ((Actigraph GT3X+)). The tracker is worn day and night. It does not need to be recharged or downloaded during the 7 days of wear. Parents will be introduced to the tracker at a clinical visit by trained staff-members and correct placement of the device onto the child will be demonstrated. In addition, body weight, body length and head circumference will be assessed at this visit.

10.3 Laboratory Tests

<u>*Maternal and paternal blood samples:*</u>

The blood samples will be analyzed for glucose, cholesterol (total, high and low density), triglyceride, insulin,

free fatty acids, amino acids, interleukin-6 and C-reactive protein at The Department of Clinical Biochemistry, NOH, and in the laboratory of Professor Mireille van Poppel at Institute of Sport Science, University of Graz in Austria. Further, blood samples will be used for epigenetic analysis, more specifically DNA methylation of PBMC's as described in detail below in the section about umbilical cord blood samples. Maternal blood samples from visit 1, 2, 3 and 4 will also be analyzed using metabolomics and lipidomics analysis techniques in the laboratory of Professor Thomas Moritz at the Novo Nordisk Foundation Center for Basic Metabolic Research at UCPH to investigate metabolites and lipids in the blood during pregnancy and delivery. The maternal blood omics data will be analyzed in relation to stress data obtained continouosly by the activity tracker during the project and in relation to other data obtained during the project (already approved). Maternal and paternal blood samples will also be analyzed for concentrations of per- and polyfluoroalkyl substances (PFAS) in the labroratory of Associate Professor Christian Lindh at Division of Occupational and Environmental Medicine, Lund University in Sweden. Maternal blood PFAS concentrations will be analyzed in relation to physical activity measures obtained by the activity tracker during the project and in relation to maternal blood lipid concentrations and to other data obtained during the project (already accepted).

The clinical laboratory values will be reported to the investigators by the laboratory and they will immediately review them for significance.

When blood samples are sent to Austria, the Act on Processing of Personal Data will be complied with.

When blood samples are sent to Sweden the General Data Protection Regulation and the Danish Data Protection Act will be complied with. A data processing agreement will be made with Lund University and excess biological material sent to Sweden will be destroyed after analyses.

Umbilical cord blood samples:

Arterial and venous blood samples will be analyzed for glucose, cholesterol (total, high and low density), triglyceride, insulin, c-peptide, free fatty acids, amino acids, adiponectin and leptin. Further, epigenetic analyses will be performed in the laboratory of Associate Professor Romain Barrès at the Novo Nordisk Foundation Center for Basic Metabolic Research, UCPH. They will perform reduced representation bisulfite sequencing on PBMC's from mother, father and offspring, in order to interrogate the DNA methylation level of approximately 2-3 million CpG's of the genome.

DNA and RNA will be recovered via AllPrep DNA/RNA/miRNA Universal Kit (Qiagen). They will use the purified DNA to clarify the DNA methylation. They will measure the entire genome's promoter DNA methylation using Multiplexed Reduced Representation Bisulfite Sequencing and Next Generation sequencing on an Illumina platform in a single read 75 base pair setup, at a depth of approximately 20 M reads per sample. The results will be verified subsequently by targeted bisulfate sequencing. Bioinformatic comparison of DNA methylomes from parents and offspring will infer on the DNA methylation marks that are transmitted to the child. Information on DNA methylomes of PBMC's from each parent will further help distinguish between paternally- and maternally-transmitted epigenetic profiles that are transmitted to the offspring. All bioinformatics analyses will be performed by the two bioinformaticians working under the supervision of Romain Barrès. Analysis will be performed in R using Bioconductor packages. Pre-processed reads of at least 15 nucleotides will be aligned to the human genome with Bismark. Differential methylation will be analyzed with methylKit. Multiple comparison correction will be done using the SLIM method. A CpG must be covered by at least ten reads in at least three samples at each time point to be included. All regions will be annotated with the bioconductor package ChIPseeker. Principal component analyses will be used to identify the specific metabolic or anthropometric features of the mother that are associated with a specific DNA methylation footprint transmitted to the offspring. All data analysis will be performed on anonymized data by the bioinformaticians.

Since it is only the DNA methylation in the maternal and paternal peripheral blood mononuclear cells and umbilical cord blood cells we are investigating, we do not conduct comprehensive sequencing of the genome, but only a smaller, targeted mapping of the methylation in CpG rich areas of the DNA Therefore, we do not

expect any risk of random genomic findings that may be of importance to the participant. We do not believe that there are currently any implications for genetic counseling of the participants in the trial. If, in anticipation, random findings of genetic sequences presenting a serious disease occur, we will relate to the principles included in the genome-application guidelines from the Ethical Committee.

Umbilical cord plasma will also be analyzed for concentrations of PFAS in the labroratory of Associate Professor Christian Lindh at Division of Occupational and Environmental Medicine, Lund University in Sweden. Umbilical cord plasma PFAS concentrations will be analyzed in relation to physical activity measures obtained by the activity tracker during the project and in relation to maternal and paternal blood PFAS concentrations and to other data obtained during the project (already accepted).

When umbilical cord blood samples are sent to Sweden the General Data Protection Regulation and the Danish Data Protection Act will be complied with. A data processing agreement will be made with Lund University and excess biological material sent to Sweden will be destroyed after analyses.

Placental samples:

Biopsies from both the maternal and fetal side of the placenta will be obtained from the placenta following birth and either snap-frozen in liquid nitrogen for down-stream processing (DNA, RNA, and protein) or fixated in paraformaldehyde for immunohistochemistry. Analysis of placental samples will be performed in the laboratory of Associate Professor Ole Hartvig Mortensen at the Department of Biomedical Sciences, UCPH and the samples will be analyzed using various methods (see below) in order to determine if exercise during pregnancy has any effect on placental function, metabolism and nutrient transport and whether or not epigenetics play a role in this. Thus, RNA and DNA will be isolated from the placental samples using AllPrep DNA/RNA universal kit (Qiagen).

The entire genome's promoter DNA methylation will be measured as described in the umbilical cord blood samples section. As in the umbilical cord blood samples, we do not conduct comprehensive sequencing of the genome, but only a smaller, targeted mapping of the methylation in CpG rich areas of the DNA Therefore, we do not expect any risk of random genomic findings that may be of importance to the participant. We do not believe that there are currently any implications for genetic counseling of the participants in the trial. If, in anticipation, random findings of genetic sequences presenting a serious disease occur, we will relate to the principles included in the genome-application guidelines from the Ethical Committee. All data analysis will be performed on anonymized data.

Global mRNA expression will be analyzed by RNA-seq which yields information regarding the number of transcripts present per mRNA. Sequencing libraries from the isolated RNA will be prepared using the NEBNext Ultra RNA Library Prep Kit for Illumina and sequenced on a HiSeqIllumina platform. The bioinformatic analysis of the RNA-seq data will be performed by Ole Hartvig Mortensen or members of his research group under his supervision. Analysis will be performed in R using Bioconductor packages. Pre-processed reads of at least 15 nucleotides will be aligned to the genome using the Rsubread package, followed by assignment of reads to genes using the featureCounts package. Statistical analysis of the read counts will be performed using the limma package to determine differences between groups and principal component analysis will be used to identify specific metabolic features of the placenta associated with training. All data analysis will be performed on anonymized data and any information regarding gene variants will be filtered out before the final analysis.

Since it is only the mRNA levels in the placenta we are investigating, we do not conduct comprehensive sequencing of the genome, but only of a smaller fraction, as only 5-10% of the genome is transcribed into RNA. Furthermore, the data-processing does not yield genetic information, but only “counts” the amount of RNA in the

end only yielding information regarding RNA expression levels. Furthermore, as the data-processing also filters out any information regarding gene variants before statistical analysis, we do not expect any risk of random genomic findings that may be of importance to the participant. We do not believe that there are currently any implications for genetic counseling of the participants in the trial. If, in anticipation, random findings of genetic sequences presenting a serious disease occur, we will relate to the principles included in the genome-application guidelines from the Ethical Committee.

Significant findings from the both the DNA methylation analysis and RNA-seq will be validated using RT-qPCR or targeted bisfulfite sequencing. We will also validate whether or not changes in mRNA levels confer changes in protein levels using western blotting where appropriate and depending on results will examine phosphorylation levels of proteins involved in signaling events in the placenta related to metabolism. Changes in protein expression levels will also be examined using immunohistochemistry as for example placement of nutrient transporters is very important. Furthermore, the general placental histology, especially membrane histology, will be examined using standard methods (e.g. Hematoxylin and Eosin staining). The metabolome of the placental samples will be examined using non-targeted NMR based metabolomics. All data analysis will be performed on anonymized data.

Placenta samples will also be analyzed for concentrations of PFAS in the labroratory of Associate Professor Christian Lindh at Division of Occupational and Environmental Medicine, Lund University in Sweden. Placental PFAS concentrations will be analyzed in relation to physical activity measures obtained by the activity tracker during the project and in relation to maternal blood, paternal blood, and umbilical cord plasma PFAS concentrations and to other data obtained during the project (already accepted).

When placenta samples are sent to Sweden the General Data Protection Regulation and the Danish Data Protection Act will be complied with. A data processing agreement will be made with Lund University and excess biological material sent to Sweden will be destroyed after analyses.

Urine samples for DLW analysis:

DLW analyses will be performed at the Clinical Metabolomics Core Facility, Rigshospitalet, which is led by Professor Gerrit van Hall. The DLW technique is based on the principle that the disappearance rate of the heavier stable isotope of hydrogen (^2H) reflects water turnover rate, whereas the disappearance rate of the heavier stable isotope of oxygen (^{18}O) reflects both water and CO_2 turnover rates. Therefore, with time, the difference between the disappearance rates of ^2H and ^{18}O represents the rate of CO_2 production. Based on the energy equivalent of CO_2, the rate of CO_2 production can be converted to total energy expenditure. Subsequently, activity-induced energy expenditure is determined by subtraction of estimates of basic metabolic rate and diet-induced energy expenditure (149). Besides estimating free-living energy expenditure, DLW will also be used to estimate maternal body composition during pregnancy from total body water determined from the post-absorptive ^2H_2O enrichments (133). Also, energy intake will be assessed via DLW (124).

Breast milk samples:

Analysis of the breast milk samples will be performed by assistant professor at The Ohio State University, Kristin Stanford. Data from her lab show that when they cross-foster mice born from sedentary moms to exercise-trained moms, they have improved glucose tolerance, body weight, and decreased adiposity at one year of age - all offspring were sedentary. The effect of drinking milk from trained moms was enough to improve their whole-body metabolism (Stanford, K., preliminary results).
Breast milk samples will also be analyzed for concentrations of PFAS in the labroratory of Associate Professor Christian Lindh at Division of Occupational and Environmental Medicine, Lund University in Sweden. Breast milk PFAS concentrations will be analyzed in relation to physical activity measures obtained by the activity tracker

during the project and in relation to maternal blood, paternal blood, and umbilical cord plasma PFAS concentrations and to other data obtained during the project (already accepted).

When breast milk samples are sent to Sweden the General Data Protection Regulation and the Danish Data Protection Act will be complied with. A data processing agreement will be made with Lund University and excess biological material sent to Sweden will be destroyed after analyses.

Validation of activity tracker for measuring sleep by polysomnography (PSG):

A one-night sleep study will be conducted in collaboration with Poul Jennum, chief physician at the Danish Center for Sleep Medicine and Professor of Neurophysiology at the University of Copenhagen, to test the validity of the activity tracker to measure sleep metrics. PSG is considered the “gold standard” for measuring sleep, and we will use ambulatory PSG in a subgroup of FitMum participants. Analysis of the PSG-data from the one-night sleep study will be performed at Professor Jennum’s clinic at Rigshospitalet – Glostrup.

Explore the activity tracker data to predict physiological outcomes

The data from activity trackers used during the FitMum study will be further explored to investigate the possibilities of predicting physiological outcomes over time in pregnant women. The activity tracker data will be investigated by using models on machine learning algorithms. This exploration of the data will be in collaboration with Jakob Eg Larsen, who is an associate professor at the Technical University of Denmark (DTU), Dept. of Applied Mathematics and Computer Science.

11. Assessment of safety

A few safety precautions are applied for the participants in the EXE and MOT groups. If a participant experiences vaginal bleeding, she will have a medical check and be temporarily paused from the intervention until not having experienced vaginal bleedings for seven consecutive days. If a participant experiences painful increased uterine activity or pain, she will have a medical check and be encouraged to appropriately reduce her physical activity level. Participants will be informed to stop physical activity and to immediately contact the Department of Gynecology and Obstetrics, NOH, if they experience any sign of preterm delivery or other pregnancy related complications.

For safety reasons, a few measurements are obtained during the trial and the following procedures are applied. An ultrasound scan is performed to confirm a living intrauterine pregnancy before inclusion in the trial. The participant’s weight will be measured at visit 1, 2 and 3, and symphysis-fundal height measurement will be obtained at visit 2 and 3. Deviating findings in symphysis-fundal height measurement and weight in relation to the Institute of Medicine’s pregnancy weight guidelines (150) will result in a medical check and a fetus growth scan will be performed for further safety investigation. Further, if a participant experiences a feeling of pelvic heaviness, flour or frequent uterine activity, she will have a medical check and a cervix scan of cervical length may be performed for further safety investigation. The participants attend the already existing pregnancy and delivery program at NOH including regular midwife consultations where they are also screened for any potential risks in relation to their pregnancies.
Participants will have easy access to sparring with the research team if there is concern about their child's well-being and psycho-motor development. Participants can take direct contact to the research team by email or phone. In case of abnormal low ASQ-3 scores, registered after parental completion of the ASQ-3 questionnaire for 12-month, parents will be contacted by an experienced pediatric nurse from the research team, and if necessary, the child will be examined by a pediatrician from Department for Children and Adolescents, NOH. No action will be taken in case of low score on the ASQ-3 14-month questionnaire, since this is only completed for research purpose (see statistical analysis).
Similarly, the extra screening for signs of postpartum depression provides extra assurance that parents will receive the necessary help and support if needed. In case of abnormal high scores on signs of post-partum depression in any of the parents, registered after parental completion of the “Parental mental well-being questionnaire”, parents will be contacted by an experienced pediatric nurse from the research team. Participants

will be offered support to establish contact to the relevant healthcare service, which could be the local healthcare nurse, the general practitioner or a psychiatric ward, depending on severity of depressive symptoms. Information about adverse events (AEs) and serious adverse events (SAEs), whether reported by the participant, discovered by the investigators by reviewing medical records, detected through examinations on visits at NOH, laboratory test or other means, occurring from the time the informed consent was signed by the participant and until visit 5 will be recorded and reported on an AE page in the electronic case report form (e-CRF). Evaluation of AEs and SAEs including severity, causality, outcome and seriousness assessments will be performed by a physician. Use of pain relieving medicine in relation to delivery is considered to be included as a natural part of the trial and will therefore not be recorded as AEs. Hospitalization in relation to delivery is considered to be included as a natural part of the trial as well, and is not recorded as a SAE.
During COVID-19 we haven't been able to measure symphysis-fundal height or weigh the women in the Fitmum study, but since the women are still followed by the midwifes at the hospital and their own doctor as before COVID-19, their safety is not compromised.

11.1 Reporting

AE and SAE reporting will be performed in the e-CRF – and reviewed by:

Contact person: Ellen Christine Leth Løkkegaard,
Professor, Chief Physician, Department of Gynecology and Obstetrics, NOH

Email address: Ellen.Christine.Leth.Loekkegaard@regionh.dk

Emergency phone: +45 4828 6249

12. Data handling / Data Management

An electronic case report form (Research Electronic Data Capture, REDCap) is provided and all data related to the trial will be recorded in here and provide the basis for a central database. In this central database data will be stored in coded form according to the rules of the Danish Data Protection Agency (Datatilsynet) with whom the trial will be registered. Personal data processing is complied with, with respect to the Act on Processing of Personal Data. The e-CRF is to be completed by the investigators at the time of the participant's visit at NOH so that it always reflects the latest observations for the participant.

Data will be stored for 25 years, after which they will be transferred to "Rigsarkivet" in an anonymized format. Source data will be registered in medical records, on source data sheets or directly in the e-CRF.

The following people will be involved in data collection and processing: Ellen Christine Leth Løkkegaard, Bente Merete Stallknecht, Jakob Eg Larsen, Romain Barrés, Ole Hartvig Mortensen, Mireille van Poppel, Gerrit van Hall, Tine Clausen, Jane Bendix, Stig Mølsted, Astrid Jespersen, Helle Terkildsen Maindal, Bodil Rasmussen, Ralph Maddison, Kristin Stanford, Thomas Moritz, Signe de Place Knudsen, Caroline Borup Roland, Saud A. Alomairah, Desirée Hornbæk, Milling, Kasper Pilgaard, Grete Teilmann, Bo Mølholm, Poul Jennum, Anne Dsane Andersen and master students doing their masters project in the setting of FitMum RCT.

13. Sample size and statistical evaluation

13.1 Sample size

Sample size has been calculated for the primary endpoint, physical activity level among the pregnant women from randomization to GA week 28+0-6. We consider a scenario with a comparison of three groups (CON, EXE and MOT). Assuming that the two interventions have an effect, we stipulate that the average activity levels for the three groups are 60, 150 and 210 min/week, respectively. The stipulation is based on the participants in the intervention groups reaching the target of being physically active for at least 30 min/day (7 x 30 min/day = 210 min/week). Also, we would like to be able to detect a difference in weekly activity level of 60 min/week. Therefore, an activity level of 150 min/week is stipulated in one of the intervention groups. Lastly, the activity level in CON is stipulated to be 60 min/week as structured exercise at moderate to vigorous intensity for more than one hour per week is an exclusion criteria. Based on a literature study (77), we estimate that the standard deviations in the three groups are the same and equal to 116 min/week. We assume that data is normally distributed. We want strength of 80%.

We consider the following three different alternative hypotheses:

1. The average activity levels in the three groups are significantly different from each other (an omnibus test)
2. The average activity levels in the two intervention groups are significantly different from each other (a pure intervention comparison)
3. The average activity levels in the three groups are significantly different from each other and the average activity levels in the two intervention groups are also significantly different from each other (a double hypothesis)

It is clear that under our assumptions, hypothesis 2 requires a larger sample size than hypothesis 1. Conversely, hypothesis 3 will also require a larger sample size than hypothesis 2 as this is based on the association of two hypotheses and to control the overall risk of a false positive result (Type 1 error), each of the two partial hypotheses must be tested at a lower level of significance than usual. For hypothesis 1 and 2, we apply a standard 5% significance level. A Bonferroni correction under hypothesis 3 then gives a significance level of 2.5% for each of the two partial hypotheses.

We calculate the required sample size by simulation using a likelihood ratio test. In addition, we consider two scenarios: a) the same number of people in each group (1:1:1 randomization) and b) the double number of people in each intervention group relative to the number in CON (2:2:1 randomization).

Ad a) Sample size needed to test hypothesis 1 is 12 participants in each of three groups. For hypothesis 2, 60 participants are needed in each group and to test hypothesis 3, 70 participants are needed in each group; 210 participants in total.

Ad b) Sample size needed to test hypothesis 1 is 9 participants in CON and 18 participants in EXE and MOT, respectively. For hypothesis 2, 30 participants are needed in CON and 60 in EXE and MOT, respectively. To test hypothesis 3 requires 35 participants in CON and 70 participants in EXE and MOT, respectively, and hence 175 participants in total (Figure 4).

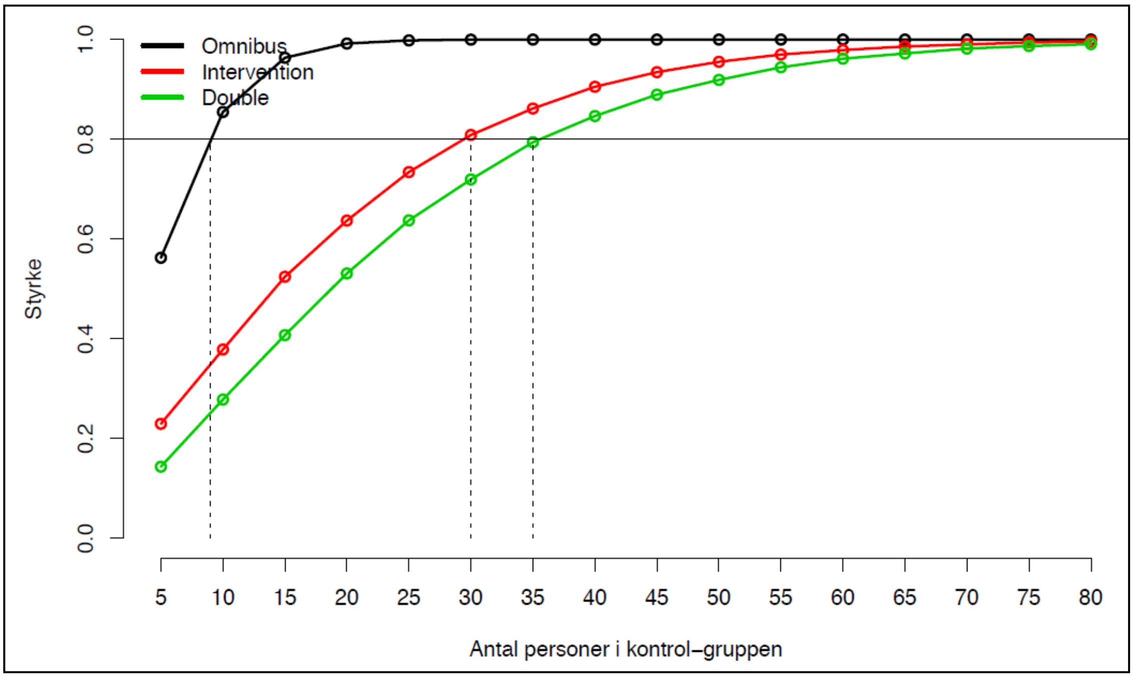

Figure 4: Overview of sample size of participants needed in the control group to test hypothesis 1 (black curve), 2 (red curve) and 3 (green curve) with a strength of 80 %, considering scenario b characterized by having the double number of people in each intervention group relative to the number in the control group (2:2:1 randomization).

We choose to randomize 2:2:1 (EXE:MOT:CON) as this requires the lowest number of participants, and gives the participants (who we assume are motivated for being more physically active) the greatest chance of being randomized to an intervention group. Thus, due to our sample size calculation above we will include 35 participants in CON and 70 participants in EXE and MOT, respectively. Further, expected loss-to-follow-up rate needs to be considered and added to the sample size. Therefore, we will include 44 participants in CON and 88 participants in EXE and MOT, respectively, in total 220 women, based on an expected loss-to-follow-up rate of 20%, as seen in similar exercise studies in pregnant women (21,23,27,77). This should leave 35 participants in CON (0.8 x 44) and 70 participants in each of the two interventions groups (0.8 x 88), which will give sufficient power for analysis of the primary endpoint.

Approximately 4,000 deliveries take place annually at the Department of Gynecology and Obstetrics, NOH. A conservative estimate would be that 1/10 of the women are eligible and willing to be included in FitMum RCT, and it should thus be possible to recruit 220 pregnant women in approximately 12 months.

13.2 Statistical analyses

Data analyses of both primary and secondary endpoints will be performed using intention-to-treat analyses (all randomized participants). Additionally, per-protocol analyses including participants not deviating from the protocol will be conducted for clinical and metabolic health parameters in the mother and child. The data analyses will be performed blinded by members of the project group in close collaboration with a statistician experienced in analyses of biomedical data. All data will be described including data-incompleteness as well as reasons for data-incompleteness. Regarding participants lost to follow-up, data obtained until trial exit will be used in the intention-to-treat analyses. Participants that are discontinued from the EXE or MOT interventions are encouraged to keep wearing the activity tracker and to attend the remaining visits at NOH in order to enable access to retest data to be used in the intention-to-treat analyses. Before the randomization code is added to

the cleaned dataset, a thorough plan for statistical analysis will be elaborated and accepted by the statistician and investigators.
Primary analysis on childhood growth, psychomotor development and physical activity will be performed among children born at term.

13.3 Interim analysis

No interim analyses will be performed on the primary and secondary endpoints. Interim analyses will only be performed on baseline data obtained before randomization.

14. End of trial

Within 90 days after the trial completion the principal investigator must inform the Ethical Committee about the completion. The result of the trial must be submitted to the Ethical Committee within 12 months.

14.1 Early termination of the trial

The principal investigator reserves the right to terminate the trial for any safety concerns. If the trial is prematurely terminated or suspended, the investigators should promptly inform the participants and ensure appropriate therapy and follow-up. Furthermore, the investigators and/or principal investigator should promptly inform the pertinent ethical committee and regulatory authorities.

14.2 Participants lost to follow-up or discontinued from interventions

The informed consent form will inform the women that they have the right to withdraw their informed consent at any time without prejudice. A participant withdrawing informed consent or having serious obstetric or medical complications leading to termination of pregnancy will be characterized as lost to follow-up.

Participants can also be discontinued from the EXE or MOT interventions. Participants <u>must</u> be discontinued from the EXE or MOT interventions under any of the following circumstances:

- If there is a risk of preterm birth (before GA week 37)
- If a cervical length below 25 mm is measured
- If serious obstetric or medical complications occur
- If, in the investigators' opinion, continuation in the interventions would be detrimental to the participant's well-being
- Occurrence of intolerable AEs as determined by the investigators and/or participant

Furthermore, participants can discontinue for personal reasons. In case of discontinuation, the participant is encouraged to keep wearing the activity tracker and to attend the remaining visits at NOH in order to enable access to retest data. If a participant is discontinued or lost to follow up, the measurements that have already been made, as well as all information the participant has provided, will be entered in the electronic database and included in the data analysis. However, a participant can ask to have all measurements and information about them deleted.

Participants who are lost to follow-up or discontinued from interventions will be invited to an individual qualitative interview immediately after discontinuation as described in section 10.2 about the qualitative project

investigating personal understandings of physical activity in everyday life of pregnant women and social, structural and cultural practices and factors influencing successful implementation of exercise during pregnancy.

In all cases, the primary reason for a participant being lost to follow-up or discontinued from intervention must be recorded in the e-CRF and in the participant's medical records, and must be handled with discretion by the investigators. Follow-up on the participant is necessary to establish whether the reason was an AE. If so, this must be reported in accordance with the appropriate procedures.

We will strive to limit the rate of women discontinuing from the interventions or lost to follow-up by being flexible in relation to scheduling of counseling sessions and visits at NOH, and offering training sessions for the EXE group at six to eight different times during the week (as described in section 15.2). Further, we aim to constantly adjust the content of sessions in the EXE intervention and the individual goal setting and action plan in the MOT intervention in order to prevent pregnancy-related complications to occur.

15. Administrative procedures

15.1 Organization

Professor, PhD, DMSc Bente Merete Stallknecht, Prorector at UCPH has taken the initiative for the trial, which is carried out in collaboration between UCPH, Technical University of Denmark, NOH, Aarhus University, University of Graz in Austria and Deakin University in Australia.

To ensure high quality research national and international collaborations have been established with respected researchers. The proficiency of the researchers involved supplements each other as they possess expertise on physical activity (Professor Bente Merete Stallknecht, Senior Researcher Stig Mølsted, Professor Mireille van Poppel), pregnancy (Professor, Chief Physician, Ellen Christine Leth Løkkegaard, Chief Physician, Associate Professor Tine Clausen, midwife Jane Bendix), clinical intervention design (Professor Helle Terkildsen Maindal, Professor Bodil Rasmussen), personalized health technology (Associate Professor Jakob Eg Larsen, Professor Ralph Maddison), biostatistics (Assistant professor Andreas Kryger Jensen and Professor Esben Budtz-Jørgensen), health economy (Associate Professor Mette Gørtz), qualitative interviews and observations (Associate Professor Astrid Jespersen), DLW (Professor Gerrit van Hall), fetal programming (Associate Professor Romain Barrès, Associate Professor Ole Hartvig Mortensen), exercise-induced adaptations to the breast milk (Assistant professor Kristin Stanford), sleep patterns during and after pregnancy (Professor, chief physician Poul Jennum) and parental mental well-being, childhood growth and development (Pediatrician, PhD Kasper Pilgaard and Associate Professor Grete Teilmann) and environmental medicine (Professor, Lisbeth E. Knudsen and Associate Professor Line Mathiesen and Associate Professor Christian Lindh).

Furthermore, key stakeholders, i.e. those who will use or are affected by the final outcome of FitMum RCT, have been identified. They include the Danish Health Authorities, the Danish Committee for Health Education, the Danish Association of Midwives, and health and care professionals in regions and municipalities. Preliminary contact and meetings have already taken place with many of these stakeholders. During the trial, a close and constructive contact to the stakeholders will be kept; hereby assuring that the knowledge and methods generated in FitMum RCT are transferred to these relevant key players in the health and care sector and paving the way for concrete actions and societal impact.

15.2 Facilities and infrastructures

The practical aspects of the trial are conducted at the Department of Gynecology and Obstetrics at NOH by the investigators, primarily Signe de Place Knudsen and Caroline Borup Roland (PhD students), a postdoc and master students affiliated with FitMum RCT. This will occur in close collaboration with Obstetricians, professor, Chief Physician, Ellen Løkkegaard, Chief Physician, Associate Professor Tine Clausen and Midwife, PhD, Jane

Bendix from the Department of Gynecology and Obstetrics, as well as with Senior Researcher, Physiotherapist, PhD, Stig Mølsted from the Department of Clinical Research, NOH.

The facilities and infrastructures at NOH in relation to carrying out the practical aspects of the trial are ideally suited to conduct the trial, including access to training and meeting facilities, rooms for examinations of health parameters at visits at NOH, as well as possibilities for having a Research Biobank and Biobank for future research for storage of biological material (maternal and paternal blood, umbilical cord blood, placental and urine samples).

In the EXE group, water and land exercise sessions will take place in the local indoor swimming pool and at the training facility at NOH, respectively. Training sessions will be offered at six to eight different times per week (two water exercise sessions and four land exercise sessions), including weekday mornings and afternoons, as well as weekends. The participants book their three weekly training sessions continuously through a doodle booking system, which is send by email and uploaded every fourth week in the private Facebook group established for all participants in the EXE intervention group. Participants are encouraged to avoid having several training sessions the same day and if possible have one-day rest between EXE training sessions. Self-organized activities are encouraged on non-training days.

Regarding the MOT group, individual and group counseling sessions will be held in the consultation room at the training facility at NOH. Individual sessions will mainly be offered continuously Monday to Friday from 8 AM to 5 PM. The group sessions will be offered in weekday late afternoons. The counselor schedules all individual and group sessions via a doodle for each participant send by email, and each participant is asked to either confirm or reject the appointment. If rejected, sessions will be rescheduled to fit into each participant's personal calendar.

15.3 The visits at Department of Gynecology and Obstetrics at NOH for measurement of secondary endpoints during the trial period, will also, as far as possible, be scheduled in connection with the women's routine visits at NOH in relation to the existing pregnancy and delivery program at NOH (e.g. midwife consultations and scans). Examinations of secondary endpoints at the visits at NOH will mainly take place at the Department of Clinical Research, NOH, where trained laboratory technicians can assist the FitMum RCT investigators in carrying out the measurements. Financing

Financial support for the conduct of FitMum RCT will be given to research accounts at Department of Biomedical Sciences, UCPH and Department of Gynecology and Obstetrics, NOH, respectively. The Independent Research Fond Denmark supports the trial with 3,877,860 DKK for PhD salary and running costs. Copenhagen Center for Health Technology and Beckett-Fonden support the trial with 2/3 PhD stipend and 75,000 DKK for purchase of activity trackers, respectively. Aase and Ejnar Danielsens Fond and Familien Hede Nielsens Fond support the trial with 100,000 DKK and 10,000 DKK for epigenetic analyses, respectively. Internal funds at NOH support the project with 75,000 DKK for running costs and 132,000 DKK for salary for two pregraduate research students. Further funding for conduct of the trial is currently applied for and will continuously be applied for from external funds. The National Committee on Health Research Ethics and the participants will be informed if new financial support is achieved. As compensation for time spent on visit days at the trial, participants are offered to keep their activity trackers after completion of trial.

15.4 Insurance

Patients will be covered according to current regulations by the law on patient-insurance and the law on appeal and compensation within the health care system.

16. Publication

FitMum RCT will provide evidence-based knowledge that can contribute to optimizing recommendations of physical activity during pregnancy in Denmark and worldwide as well as new, health-technology-supported exercise programs to pregnant women that are effective and simple to implement. The knowledge and tools can be transformed into initiatives in municipalities and hospitals, and the trial therefore has a great potential to improve the health and quality of life of both mother and child and to prevent the development of lifestyle-related diseases across generations.

The dissemination of the results, methods and tools of FitMum RCT are mainly targeted our key stakeholders, the international research community as well as international policymakers and practitioners. Regarding dissemination to the international research community, positive, negative and in-conclusive trial results will be published in national and international peer-reviewed scientific journals by the investigators of the project group; Caroline Borup Roland, Signe de Place Knudsen and Saud A. Alomairah (PhD students) will soon draft the first manuscript, which will describe the study design. The results have the potential to be published in highly rated international peer reviewed journals, e.g. Obstetrics & Gynecology (The Green Journal) and American Journal of Obstetrics and Gynecology.

17. References

1. Barrès R, Zierath JR. The role of diet and exercise in the transgenerational epigenetic landscape of T2DM. Nat Rev Endocrinol. 2016;12(8):441–51.

2. Adamo KB, Ferraro ZM, Brett KE. Can we modify the intrauterine environment to halt the intergenerational cycle of obesity? Int J Environ Res Public Health. 2012;9(4):1263–307.

3. Committee on Obstetric Practice. Committee Opinion: Physical Activity and Exercise During Pregnancy and the Postpartum Period. Am Coll Obstet Gynecol. 2015;650:1–8.

4. Harris JE, Baer LA, Stanford KI. Maternal Exercise Improves the Metabolic Health of Adult Offspring. Trends Endocrinol Metab. 2018;1–14.

5. World Health Organisation (WHO). Good Maternal Nutrition. The best start in life. 2016;

6. The Danish Health Authorities. Recommendations for pregnant women [Internet]. 2014. Available from: https://www.sst.dk/en/health-and-lifestyle/physical-activity/recommendations/pregnant-women

7. Broberg L, Ersbøll AS, Backhausen MG, Damm P, Tabor A, Hegaard HK. Compliance with national recommendations for exercise during early pregnancy in a Danish cohort. BMC Pregnancy Childbirth. 2015;15:317.

8. Borodulin K, Evenson KR, Herring AH. Physical activity patterns during pregnancy through postpartum. BMC Womens Health. 2009;9(1):32.

9. Santo EC, Forbes PW, Oken E, Belfort MB. Determinants of physical activity frequency and provider advice during pregnancy. 2017;1–11.

10. Hegaard HK, Damm P, Hedegaard M, Henriksen TB, Ottesen B, Dykes AK, et al. Sports and leisure time physical activity during pregnancy in nulliparous women. Matern Child Health J. 2011;15(6):806–13.

11. Owe KM, Nystad W, Bø K. Correlates of regular exercise during pregnancy: The Norwegian Mother and Child Cohort Study. Scand J Med Sci Sport. 2009;19(5):637–45.

12. Richardsen KR, Falk RS, Jenum AK, Mørkrid K, Martinsen EW, Ommundsen Y, et al. Predicting who fails to meet the physical activity guideline in pregnancy: A prospective study of objectively recorded physical activity in a population-based multi-ethnic cohort. BMC Pregnancy Childbirth. 2016;16(1):1–11.

13. Statistik D. Fødsler [Internet]. 2018. Available from: https://www.dst.dk/da/Statistik/emner/befolkning-og-valg/foedsler/foedsler

14. Ruiz JR, Perales M, Pelaez M, Lopez C, Lucia A, Barakat R. Supervised exercise-based intervention to prevent excessive gestational weight gain: A randomized controlled trial. Mayo Clin Proc. 2013;88(12):1388–97.

15. Leung Hui A, Back L, Ludwig S, Gardiner P, Sevenhuysen G, Dean HJ, et al. Effects of lifestyle intervention on dietary intake, physical activity level, and gestational weight gain in pregnant women with different pre-pregnancy Body Mass Index in a randomized control trial. BMC Pregnancy Childbirth. 2014;14(331):1–9.

16. Wiebe HW, Boulé NG, Chari R, Davenport MH. The Effect of Supervised Prenatal Exercise on Fetal Growth. Obstet Gynecol. 2015;125(5):1185–94.

17. Poston L, Bell R, Croker H, Flynn AC, Godfrey KM, Goff L, et al. Effect of a behavioural intervention in obese pregnant women (the UPBEAT study): a multicentre, randomised controlled trial. Lancet Diabetes Endocrinol. 2015;3(10):767–77.

18. Harrison CL, Lombard CB, Strauss BJ, Teede HJ. Optimizing healthy gestational weight gain in women at high risk of gestational diabetes: A randomized controlled trial. Obesity. 2013;21(5):904–9.

19. Vinter CA, Jensen DM, Ovesen P, Beck-nielsen H, Jørgensen JS. The LiP (Lifestyle in Pregnancy) Study. Diabetes Care. 2011;34:2502–7.

20. Renault KM, Nørgaard K, Nilas L, Carlsen EM, Cortes D, Pryds O, et al. The Treatment of Obese Pregnant Women (TOP) study: A randomized controlled trial of the effect of physical activity intervention assessed by pedometer with or without dietary intervention in obese pregnant women. Am J Obstet Gynecol. 2014;210:134.e1-9.

21. Backhausen MG, Tabor A, Albert H, Rosthøj S, Damm P, Hegaard HK. The effects of an unsupervised water exercise program on low back pain and sick leave among healthy pregnant women - A randomised controlled trial. PLoS One. 2017;12(9):e0182114.

22. Russo LM, Nobles C, Ertel KA, Chasan-Taber L, Whitcomb BW. Physical Activity Interventions in Pregnancy and Risk of Gestational Diabetes Mellitus A Systematic Review and Meta-analysis. Obstet Gynecol. 2015;125(3):576–82.

23. Wang C, Wei Y, Zhang X, Zhang Y, Xu Q, Sun Y, et al. A randomized clinical trial of exercise during pregnancy to prevent gestational diabetes mellitus and improve pregnancy outcome in overweight and obese pregnant women. Am J Obstet Gynecol. 2017;1–30.

24. Di Mascio D, Magro-Malosso ER, Saccone G, Marhefka GD, Berghella V. Exercise during pregnancy in normal-weight women and risk of preterm birth: a systematic review and meta-analysis of randomized controlled trials. American Journal of Obstetrics and Gynecology. 2016. p. 561–71.

25. Sanabria-Martinez G, Garcia-Hermoso A, Poyatos-Leon R, Alvarez-Bueno C, Sanchez-Lopez M, Martinez-Vizcaino V. Effectiveness of physical activity interventions on preventing gestational diabetes mellitus and excessive maternal weight gain: A meta-analysis. BJOG An Int J Obstet Gynaecol. 2015;122(9):1167–74.

26. Koivusalo SB, Rönö K, Klemetti MM, Roine RP, Lindström J, Erkkola M, et al. Gestational Diabetes Mellitus Can Be Prevented by Lifestyle Intervention: The Finnish Gestational Diabetes Prevention Study (RADIEL): A Randomized Controlled Trial. Diabetes Care. 2016;39(1):24–30.

27. Barakat R, Perales M, Cordero Y, Bacchi M, Mottola MF. Influence of Land or Water Exercise in Pregnancy on Outcomes: A Cross-sectional Study. Med Sci Sports Exerc. 2017;49(7):1397–403.

28. Huang L, Fan L, Ding P, He Y-H, Xie C, Niu Z, et al. Maternal exercise during pregnancy reduces the

risk of preterm birth through the mediating role of placenta. J Matern Neonatal Med. 2017;7058(September):1–21.

29. Magro-Malosso ER, Saccone G, Di Mascio D, Marhefka GD, Berghella V. Exercise during pregnancy risk of preterm birth in overweight and obese women: A systematic review and meta-analysis of randomized controlled trials. Acta Obstet Gynecol Scand. 2017;215(5):561–71.

30. Sundhed.dk [Internet]. 2018. Available from: https://www.sundhed.dk/borger/guides/sundhedstilbud/?SearchTerm=gravid træning&searchType=searchTerm&Page=1&PageSize=10&Tilbudstype=Graviditet og barsel&Region=-1&Udbyder=-1

31. Evenson KR, Barakat R, Brown WJ, Dargent-Molina P, Haruna M, Mikkelsen EM, et al. Guidelines for Physical Activity during Pregnancy: Comparisons From Around the World. Am J Lifestyle Med. 2014;8(2):102–21.

32. Perales M, Artal R LA. Exercise During Pregnancy. JAMA. 2017;317(11):1113–4.

33. Moyer C, Reoyo OR, May L. The Influence of Prenatal Exercise on Offspring Health: A Review. 2016;9:37–42.

34. Pomeroy J, Renström F, Gradmark AM, Mogren I, Persson M, Bluck L, et al. Maternal physical activity and insulin action in pregnancy and their relationships with infant body composition. Diabetes Care. 2013;36(2):267–9.

35. Harrod CS, Chasan-Taber L, Reynolds RM, Fingerlin TE, Glueck DH, Brinton JT, et al. Physical Activity in Pregnancy and Neonatal Body Composition: The Healthy Start Study. Obs Gynecol. 2014;124(1):257–64.

36. Clapp JF. Influence of Endurance Exercise and Diet on Human Placental Development and Fetal Growth. Placenta. 2006;27(6–7):527–34.

37. Jackson MR, Gott P, Lye SJ, Ritchie JW, Clapp JF. The effects of maternal aerobic exercise on human placental development: placental volumetric composition and surface areas. Placenta. 1995;16(2):179–91.

38. Hoang Nguyen PT, Binns CW, Nguyen CL, Van Ha AV, Chu KT, Van Duong D, et al. Physical activity during pregnancy is associated with improved breastfeeding outcomes: A prospective cohort study. Int J Environ Res Public Health. 2019;16(10).

39. Carter LG, Qi NR, Cabo R, Pearson KJ. Maternal exercise improves insulin sensitivity in mature rat offspring. 2013;45(5):832–40.

40. Stanford KI, Takahashi H, So K, Alves-Wagner AB, Prince NB, Lehnig AC, et al. Maternal Exercise Improves Glucose Tolerance in Female Offspring. 2017. 1–37 p.

41. Ribeiro TA, Tófolo LP, Martins IP, Pavanello A, de Oliveira JC, Prates KV, et al. Maternal low intensity physical exercise prevents obesity in offspring rats exposed to early overnutrition. Sci Rep. 2017;7(1):7634.

42. Polańska K, Muszyński P, Sobala W, Dziewirska E, Merecz-Kot D, Hanke W. Maternal lifestyle during pregnancy and child psychomotor development-Polish Mother and Child Cohort study. 2015;

43. Marie A, Jukic Z, Lawlor DA, Juhl M, Owe KM, Lewis B, et al. Physical activity during pregnancy and language development in the offspring.

44. Niño Cruz GI, Ramirez Varela A, da Silva ICM, Hallal PC, Santos IS. Physical activity during pregnancy and offspring neurodevelopment: A systematic review. Paediatr Perinat Epidemiol. 2018 Jul 1;32(4):369–79.

45. Carson V, Lee E-Y, Hewitt L, Jennings C, Hunter S, Kuzik N, et al. Systematic review of the relationships between physical activity and health indicators in the early years (0-4 years).

46. Jia Z, Zhang J, Trindade D, Sobko T. Physical Activity Patterns and Correlates of 9-Month-Old Chinese Infants in the Macau Population. Matern Child Health J. 2018 Oct 9;22(10):1526–33.

47. Barakat R, Pelaez M, Lopez C, Montejo R, Coteron J. Exercise during pregnancy reduces the rate of cesarean and instrumental deliveries: results of a randomized controlled trial. J Matern Neonatal Med. 2012;25(11):2372–6.

48. Nielsen EN, Andersen PK, Hegaard HK, Juhl M. Mode of Delivery according to Leisure Time Physical Activity before and during Pregnancy: A Multicenter Cohort Study of Low-Risk Women. J Pregnancy. 2017;2017:1–8.

49. Dumith SC, Domingues MR, Mendoza-Sassi R a, Cesar J a. Physical activity during pregnancy and its association with maternal and child health indicators. Rev Saude Publica. 2012;46(2):327–33.

50. Owe KM, Nystad W, Stigum H, Vangen S, Bø K. Exercise during pregnancy and risk of cesarean delivery in nulliparous women: a large population-based cohort study Katrine. 2016;00(December 2016):1–6.

51. Ferrari N, Bae-Gartz I, Bauer C, Janoschek R, Koxholt I, Mahabir E, et al. Exercise during pregnancy and its impact on mothers and offspring in humans and mice. J Dev Orig Health Dis. 2017;1–14.

52. Barakat R, Cordero Y, Coteron J, Luaces M, Montejo R. Exercise during pregnancy improves maternal glucose screen at 24-28 weeks: a randomised controlled trial. 2012;46:656–61.

53. Butler CL, Williams MA, Sorensen TK, Frederick IO, Leisenring WM. Relation between maternal recreational physical activity and plasma lipids in early pregnancy. Am J Epidemiol. 2004;160(4):350–9.

54. Van Poppel MNM, Peinhaupt M, Eekhoff MEW, Heinemann A, Oostdam N, Wouters MGAJ, et al. Physical activity in overweight and obese pregnant women is associated with higher levels of proinflammatory cytokines and with reduced insulin response through interleukin-6. Diabetes Care. 2014;37(4):1132–9.

55. Sagedal LR, Sanda B, Øverby NC, Bere E, Torstveit MK, Lohne-Seiler H, et al. The effect of prenatal lifestyle intervention on weight retention 12 months postpartum: results of the Norwegian Fit for Delivery randomised controlled trial. BJOG An Int J Obstet Gynaecol. 2017;124(1):111–21.

56. Tinius R, Cahill A, Cade W. Low-intensity Physical Activity is Associated woth Lower Maternal Systemic Inflammation during Late Pregnancy. J Obes Weight Loss Ther. 2017;7(3):1–8.

57. Madsen SA et al. Tidlig opsporing af fødselsdepression hos vorden-ende mødre og faedre [Internet]. 2017 [cited 2019 Apr 11]. Available from: https://www.rigshospitalet.dk/presse-og-nyt/nyheder/nyheder/Documents/JMC - dokumenter til nyheder/Screening for fødselsdepression under graviditet RESULTATER.pdf

58. Roshaidai S, Arifin M, Cheyne H, Maxwell M. Review of the prevalence of postnatal depression across cultures. AIMS Public Heal [Internet]. 2018 [cited 2019 Apr 11];5(3):260–95. Available from: http://www.aimspress.com/journal/aimsph

59. Banti S, Mauri M, Oppo A, Borri C, Rambelli C, Ramacciotti D, et al. From the third month of pregnancy to 1 year postpartum. Prevalence, incidence, recurrence, and new onset of depression. Results from the Perinatal Depression–Research & Screening Unit study. 2011 [cited 2019 Apr 11]; Available from: www.elsevier.com/locate/comppsych

60. Norhayati MN, Nik Hazlina NH, Asrenee AR, Emilin WMAW, Perpustakaan D, Tahir H. Magnitude and risk factors for postpartum symptoms: A literature review. 2014 [cited 2019 Apr 11]; Available from: http://dx.doi.org/10.1016/j.jad.2014.12.041

61. Letourneau NL, Tramonte L, Willms JD. Maternal Depression, Family Functioning and Children's Longitudinal Development. J Pediatr Nurs [Internet]. 2013 May [cited 2019 Apr 11];28(3):223–34. Available from: http://www.ncbi.nlm.nih.gov/pubmed/22940454

62. Daley AJ, Foster L, Long G, Palmer C, Robinson O, Walmsley H, et al. The effectiveness of exercise for the prevention and treatment of antenatal depression: Systematic review with meta-analysis. BJOG An Int J Obstet Gynaecol. 2015;122(1):57–62.

63. Pritchett RV, Daley AJ, Jolly K. Does aerobic exercise reduce postpartum depressive symptoms? a systematic review and meta-analysis. Br J Gen Pract [Internet]. 2017 Oct [cited 2019 Apr 11];67(663):e684–91. Available from: http://www.ncbi.nlm.nih.gov/pubmed/28855163

64. Davenport; AMBS, Boulé NG, Sivak A, Davenport MH. Effects of Exercise on Mild-to-moderate Depressive Symptoms in the Postpartum Period. Obstet &amp [Internet]. 2017 Jun 1 [cited 2019 Apr 11];129(6):1087–97. Available from: http://www.ncbi.nlm.nih.gov/pubmed/28486363

65. Nakamura A, van der Waerden J, Melchior M, Bolze C, El-Khoury F, Pryor L. Physical activity during pregnancy and postpartum depression: Systematic review and meta-analysis. J Affect Disord [Internet]. 2019 Mar 1 [cited 2019 Jan 21];246:29–41. Available from: https://www.sciencedirect.com/science/article/pii/S0165032718318779

66. Teychenne M, York R. Physical Activity, Sedentary Behavior, and Postnatal Depressive Symptoms A Review. Am J Prev Med [Internet]. 2013 [cited 2019 Apr 11];45(2):217–27. Available from: http://dx.doi.org/10.1016/j.amepre.2013.04.004

67. Thangaratinam S, Rogozińska E, Jolly K, Glinkowski S, Duda W, Borowiack E, et al. Interventions to reduce or prevent obesity in pregnant women: A systematic review. Health Technol Assess (Rockv). 2012;16(31):1–8.

68. Jelsma JGM, Van Leeuwen KM, Oostdam N, Bunn C, Simmons D, Desoye G, et al. Beliefs, Barriers, and Preferences of European Overweight Women to Adopt a Healthier Lifestyle in Pregnancy to Minimize Risk of Developing Gestational Diabetes Mellitus: An Explorative Study. J Pregnancy. 2016;1–11.

69. Hill B, McPhie S, Moran LJ, Harrison P, Huang TTK, Teede H, et al. Lifestyle intervention to prevent obesity during pregnancy: Implications and recommendations for research and implementation. Midwifery. 2017;49(May 2016):13–8.

70. Bauer C, Graf C, Platschek AM, Strüder HK, Ferrari N. Reasons, Motivational Factors and Percieved Personal Barriers to Engagement in Physical Activity During Pregnancy Vary Within the BMI Classes - The Prenatal Prevention Project Germany. J Phys Act Heal. 2017;32:1–44.

71. Weir Z, Bush J, SC R, McParlin C, Rankin J, Bell R. Physical activity in pregnancy: a qualitative study of the beliefs of overweight and obese pregnant women. BMC Pregnancy Childbirth. 2010;10(18):1–7.

72. Hopkinson Y, Hill DM, Fellows L, Fryer S. Midwives understanding of physical activity guidelines during pregnancy. Midwifery. 2018;59:23–6.

73. Lindqvist M, Persson M, Mogren I. "Longing for individual recognition" – Pregnant women's experiences of midwives' counselling on physical activity during pregnancy. Sex Reprod Healthc. 2018;15:46–53.

74. Kong KL, Campbell CG, Foster RC, Peterson AD, Lanningham-Foster L. A pilot walking program promotes moderate-intensity physical activity during pregnancy. Med Sci Sports Exerc. 2014;462–71.

75. Smith KM. The Blossom Project Online: Use of a behaviorally-based website to promote physical activity and prevent excessive gestational weight gain in previously sedentary pregnant women. ProQuest Dissertations and Theses. 2014.

76. Hawkins M, Chasan-Taber L, Marcus B, Stanek E, Braun B, Ciccolo J, et al. Impact of an exercise

intervention on physical activity during pregnancy: The behaviors affecting baby and you study. Am J Public Health. 2014;104(10):e74-81.

77. Oostdam N, Van Poppel MNM, Wouters MGAJ, Eekhoff EMW, Bekedam DJ, Kuchenbecker WKH, et al. No effect of the FitFor2 exercise programme on blood glucose, insulin sensitivity, and birthweight in pregnant women who were overweight and at risk for gestational diabetes: Results of a randomised controlled trial. BJOG An Int J Obstet Gynaecol. 2012;119:1098–107.

78. Seneviratne S, Jiang Y, Derraik J, Mccowan L, Parry G, Biggs J, et al. Effects of antenatal exercise in overweight and obese pregnant women on maternal and perinatal outcomes: a randomised controlled trial. BJOG. 2016;123:588–97.

79. Ussher M, Lewis S, Aveyard P, Manyonda I, West R, Lewis B, et al. Physical activity for smoking cessation in pregnancy: randomised controlled trial. BMJ. 2015;350(h2145):1–10.

80. Gaston A, Prapavessis H. Using a combined protection motivation theory and health action process approach intervention to promote exercise during pregnancy. J Behav Med. 2014;37(2):173–84.

81. Vinter CA, Jensen DM, Ovesen P, Beck-Nielsen H, Jørgensen JS. The LiP (Lifestyle in Pregnancy) study: A randomized controlled trial of lifestyle intervention in 360 obese pregnant women. Diabetes Care. 2011;34:2502–7.

82. Cavalcante SR, Cecatti JG, Pereira RI, Baciuk EP, Bernardo AL, Silveira C. Water aerobics II: maternal body composition and perinatal outcomes after a program for low risk pregnant women. Reprod Health. 2009;6(1):1–7.

83. Haakstad L, Bo K. Exercise in pregnant women and birth weight: a randomized controlled trial. BMC Pregnancy Childbirth. 2011;11(1):1–7.

84. Tomic V, Sporis G, Tomic J, Milanovic Z, Zigmundovac-Klaic D, Pantelic S. The effect of maternal exercise during pregnancy on abdominal fetal growth. Croat Med J. 2013;54:362–8.

85. De Oliveria Melo AS, Silva JLP, Tavares JS, Barros VO, Leite DFB, Amorim MMR. Effect of a physical exercise program during pregnancy on uteroplacental and fetal blood flow and fetal growth: A randomized controlled trial. Obstet Gynecol. 2012;120(2 PART 1):302–10.

86. Currie S, Sinclair M, Murphy MH, Madden E, Dunwoody L, Liddle D. Reducing the Decline in Physical Activity during Pregnancy: A Systematic Review of Behaviour Change Interventions. PLoS One. 2013;8(6):1–12.

87. Pearce E, Evenson K. Strategies to promote physical activity during pregnancy. Am J Lifestyle Med. 2013;7(1):1–19.

88. UK Chief Medical Officers. UK Chief Medical Officers Recommendations 2017: Physical Activity in Pregnancy. 2017.

89. UK Chief Medical Officers. UK Chief Medical Officers Recommendations 2017: Physical Activity in Pregnancy, guidance. 2017;1–7.

90. Aparicio VA, Ocón O, Padilla-Vinuesa C, Soriano-Maldonado A, Romero-Gallardo L, Borges-Cósic M, et al. Effects of supervised aerobic and strength training in overweight and grade I obese pregnant women on maternal and foetal health markers: the GESTAFIT randomized controlled trial. BMC Pregnancy Childbirth. 2016;16(290):1–13.

91. O'Connor PJ, Poudevigne MS, Cress ME, Motl RW, Clapp JF. Safety and Efficacy of Supervised Strength Training Adopted in Pregnancy. J Phys Act Heal. 2011;8(3):309–20.

92. Hayes L, Mcparlin C, Kinnunen TI, Poston L, Robson SC, Bell R. Change in level of physical activity during pregnancy in obese women: findings from the UPBEAT pilot trial. BMC Pregnancy Childbirth.

2015;15(52):1–8.

93. Maher C, Ryan J, Ambrosi C, Edney S. Users' experiences of wearable activity trackers: a cross-sectional study. BMC Public Heal 2017 171. 2017;17(880):1–8.

94. De Vries HJ, Kooiman TJM, van Ittersum MW, van Brussel M, de Groot M. Do activity monitors increase physical activity in adults with overweight or obesity? A systematic review and meta-analysis. Obesity. 2016;24(10):2078–91.

95. Obling KH, Overgaard K, Juul L, Maindal HT. The MILE study: a motivational, individual and locally anchored exercise intervention among 30-49 year-olds with low levels of cardiorespiratory fitness: a randomised controlled study in primary care. BMC Public Health. 2013;13:1224.

96. Duda JL, Williams GC, Ntoumanis N, Daley A, Eves FF, Mutrie N, et al. Effects of a standard provision versus an autonomy supportive exercise referral programme on physical activity, quality of life and well-being indicators: a cluster randomised controlled trial. Int J Behav Nutr Phys Act. 2014;11(10):1–15.

97. Rouse PC, Veldhuijzen Van Zanten JJ, Metsios GS, Ntoumanis N, Yu C, Koutedakis Y, et al. Fostering autonomous motivation, physical activity and cardiorespiratory fitness in rheumatoid arthritis: protocol and rationale for a randomised control trial. BMC Musculoskelet Disord. 2014;15(445):1–9.

98. Esbensen BA, Thomsen T, Hetland ML, Beyer N, Midtgaard J, Løppenthin K, et al. The efficacy of motivational counseling and SMS-reminders on daily sitting time in patients with rheumatoid arthritis: protocol for a randomized controlled trial. Trials. 2015;16(23):1–12.

99. Sundheds- og ældreministeriet. En god og sikker start på livet: Målsætninger for fremtidens fødselsindsats. 2018;

100. Markland D, Ryan RM, Tobin VJ, Rollnick S. Motivational Interviewing and Self–Determination Theory. J Soc Clin Psychol. 2005;24(6):811–31.

101. Michie S, Ashford S, Sniehotta FF, Dombrowski SU, Bishop A, French DP. A refined taxonomy of behaviour change techniques to help people change their physical activity and healthy eating behaviours: The CALO-RE taxonomy. Psychol Heal. 2011;26(11):1479–98.

102. Cadmus-bertram LA, Marcus BH, Patterson RE, Parker BA, Morey BL. Randomized Trial of a Fitbit-Based Physical Activity Intervention for Women. Am J Prev Med. 2015;49(3):414–8.

103. Redman LM, Gilmore LA, Breaux J, Thomas DM, Elkind-Hirsch K, Stewart T, et al. Effectiveness of SmartMoms, a Novel eHealth Intervention for Management of Gestational Weight Gain: Randomized Controlled Pilot Trial. JMIR mHealth uHealth. 2017;5(9):1–8.

104. Lobelo F, Kelli HM, Tejedor SC, Pratt M, McConnell M V., Martin SS, et al. The Wild Wild West: A Framework to Integrate mHealth Software Applications and Wearables to Support Physical Activity Assessment, Counseling and Interventions for Cardiovascular Disease Risk Reduction. Prog Cardiovasc Dis. 2016;58(6):584–94.

105. Kooiman TJM, Dontje ML, Sprenger SR, Krijnen WP, van der Schans CP, de Groot M. Reliability and validity of ten consumer activity trackers. BMC Sports Sci Med Rehabil. 2015;7(24):1–11.

106. Dontje ML, De Groot M, Lengton RR, Van Der Schans CP, Krijnen WP. Measuring steps with the Fitbit activity tracker : an inter - device reliability study. J Med Eng Technol. 2015;39(5):1464–522.

107. Wright SP, Hall Brown TS, Collier SR, Sandberg K. How consumer physical activity monitors could transform human physiology research. Am J Physiol - Regul Integr Comp Physiol. 2017;312:R358–67.

108. Foley L, Maddison R, Jones Z, Brown P, Davys A. Comparison of two modes of delivery of an exercise prescription scheme. N Z Med J. 2011;124(1338):44–54.

109. Verdiere S, Guinhouya BC, Salerno D, Deruelle P. Should physical activity be contraindicated during pregnancy in relation to its potentially related risks? 2017;45:104–11.

110. Melo ASDO, Silva JLP, Tavares JS, Barros VO, Leite DFB, Amorim MMR. Effect of a Physical Exercise Program During Pregnancy on Uteroplacental and Fetal Blood Flow and Fetal Growth. 2012;120(2):302–10.

111. Rêgo AS, Alves MTSS de B e, Batista RFL, Ribeiro CCC, Bettiol H, Cardoso VC, et al. Physical activity in pregnancy and adverse birth outcomes. Cad Saude Publica. 2016;32(11):1–10.

112. Charlesworth S, Foulds HJ a, Burr J, Bredin SSD. Evidence-based risk assessment and recommendations for physical activity clearance: pregnancy. Appl Physiol Nutr Metab. 2011;36(S1):S154–89.

113. Pitchford EA, Ketcheson LR, Kwon H-J, Ulrich DA. Minimum Accelerometer Wear Time in Infants: A Generalizability Study. J Phys Act Heal [Internet]. 2017 Jun 7 [cited 2019 Apr 11];14(6):421–8. Available from: http://journals.humankinetics.com/doi/10.1123/jpah.2016-0395

114. Prioreschi A, Nappey T, Westgate K, Olivier P, Brage S, Micklesfield LK. Development and feasibility of a wearable infant wrist band for the objective measurement of physical activity using accelerometery. [cited 2019 Apr 11]; Available from: https://doi.org/10.1186/s40814-018-0256-x

115. Katz VL. Exercise in Water During Pregnancy. Clin Obstet Gynecol. 2003;46(2):432–41.

116. Juhl M, Kogevinas M, Andersen PK, Andersen A-MN, Olsen J. Is Swimming During Pregnancy a Safe Exercise? Epidemiology. 2010;21(2):253–8.

117. Borg GAV. Psychophysical bases of perceived exertion. 1982.

118. Santini De Oliveira C, Dos Santos Imakawa T, Christine Dantas Moises E. Physical Activity during Pregnancy: Recommendations and Assessment Tools. Rev Bras Ginecol Obs. 2017;39:424–32.

119. Chasan-Taber L, Schmidt MD, Roberts DE, Hosmer D, Markenson G, Freedson PS. Development and validation of a pregnancy physical activity questionnaire. Med Sci Sports Exerc. 2004;36(10):1750–60.

120. Schuster S, Sklempe Kokic I, Sindik J. Measuring Physical Activity in Pregnancy Using Questionnaires: a Meta-Analysis. Acta Clin Croat. 2016;55(3):440–51.

121. Abeysekera M V, Morris JA, O ’sullivan AJ. Techniques to measure free-living energy expenditure during pregnancy – A guide for clinicians and researchers. Obstet Med. 2014;7(2):60–5.

122. Van Hees VT, Renström F, Wright A, Gradmark A, Catt M, Chen KY, et al. Estimation of daily energy expenditure in pregnant and Non-Pregnant women using a Wrist-Worn Tri-Axial accelerometer. PLoS One. 2011;6(7):1–10.

123. Lof M, Forsum E. Activity pattern and energy expenditure due to physical activity before and during pregnancy in healthy Swedish women. Br J Nutr. 2006;95(2):296–302.

124. Wong WW, Roberts SB, Racette SB, Das SK, Redman LM, Rochon J, et al. The Doubly Labeled Water Method Produces Highly Reproducible Longitudinal Results in. J Nutr. 2014;144:777–83.

125. Moore GF, Audrey S, Barker M, Bond L, Bonell C, Hardeman W, et al. Process evaluation of complex interventions: Medical Research Council guidance. BMJ. 2015 Mar;350:h1258.

126. Hoffmann TC, Erueti C, Glasziou PP. Poor description of non-pharmacological interventions: Analysis of consecutive sample of randomised trials. BMJ. 2013;347(7924):1–10.

127. Craig P, Dieppe P, Macintyre S, Michie S, Nazareth I, Petticrew M. Developing and evaluating complex interventions: the new Medical Research Council guidance. BMJ. 2008;337(a1655):1–6.

128. Craig P. Developing and evaluating complex interventions.

129. Moore G, Audrey S, Barker M, Bond L, Bonell C, Cooper C, et al. Process evaluation in complex public health intervention studies: the need for guidance. J Epidemiol Community Health. 2014 Feb;68(2):101–2.

130. F. Moore G, Raisanen L, Moore L, Ud Din N, Murphy S. Mixed-method process evaluation of the Welsh National Exercise Referral Scheme. Health Educ. 2013 Oct;113(6):476–501.

131. Dybboe Bjerre E, Blaedel A, Hansen G. Komplekse interventioner i medicinsk forskning.

132. Oakley A, Strange V, Bonell C, Allen E, Stephenson J, RIPPLE Study Team. Process evaluation in randomised controlled trials of complex interventions. BMJ. 2006 Feb;332(7538):413–6.

133. van Raaij JM, Peek ME, Vermaat-Miedema SH, Schonk CM, Hautvast JG. New equations for estimating body fat mass in pregnancy from body density or total body water. Am J Clin Nutition. 1988;48:24–9.

134. RAND Corporation. 36-Item Short Form Survey Instrument (SF-36). 2016;5–10.

135. Health Assessment Lab, Medical Outcomes Trust and QualityMetric Incorporated. SF-36v2 Health Survey. 2004;

136. Jomeen J, Martin CR. The factor structure of the SF-36 in early pregnancy. J Psychosom Res. 2005 Sep;59(3):131–8.

137. Cox JL (John L, Holden J. Perinatal mental health : a guide to the Edinburgh Postnatal Depression Scale (EPDS). Gaskell; 2003. 125 p.

138. Zierau F, Bille A, Rutz W, Bech P. The Gotland Male Depression Scale: A validity study in patients with alcohol use disorder. Nord J Psychiatry [Internet]. 2002 Jan 12 [cited 2019 Apr 11];56(4):265–71. Available from: http://www.tandfonline.com/doi/full/10.1080/08039480260242750

139. Magovcevic M, Addis ME. The Masculine Depression Scale: Development and psychometric evaluation. Psychol Men Masc [Internet]. 2008 Jul [cited 2019 Apr 11];9(3):117–32. Available from: http://doi.apa.org/getdoi.cfm?doi=10.1037/1524-9220.9.3.117

140. University of Pittsburgh. Pittsburgh Sleep Quality Index (PSQI). 1988;(3):6.

141. Buysse DJ, Reynolds CF, Monk TH, Berman SR, Kupfer DJ. The Pittsburgh Sleep Quality Index : A New Instrument Psychiatric Practice and Research. Psychiatry Res. 1989;28:193–213.

142. Qiu C, Gelaye B, Zhong Q-Y, Enquobahrie DA, Frederick IO, Williams MA. Construct validity and factor structure of the Pittsburgh Sleep Quality Index among pregnant women in a Pacific-Northwest cohort. Sleep Breath. 2016;20(1):293–301.

143. Rigshospitalet - Glostrup, Neurofysiologisk Klinik. Polysomnografi udført i eget hjem [Internet]. [cited 2019 Apr 11]. Available from: www.rigshospitalet.dk/dcsm

144. Bland HW, Melton BF, Marshall ES, Nagle JA. Measuring Exercise Self-Efficacy in Pregnant Women: Psychometric Properties of the Pregnancy-Exercise Self-Efficacy Scale (P-ESES). J Nurs Meas. 2013 Dec 1;21(3):349–59.

145. Mullan E, Markland D, Ingledew DK. A graded conceptualisation of self-determination in the regulation of exercise behaviour: Development of a measure using confirmatory factor analytic procedures. Vol. 23, ~) Pergamon Person. individ. Diff. 1997.

146. Mullan E, Markland D, Mullan E. Variations in Self-Determination Across the Stages of Change for Exercise in Adults. Vol. 21, Motivation and Emotion. Markland & Hardy; 1997.

147. Markland D, Tobin V. A Modification to the Behavioural Regulation in Exercise Questionnaire to Include

an Assessment of Amotivation. J Sport Exerc Psychol. 2004 Jun;26(2):191–6.

148. Plomgaard AM, Hansen BM, Greisen G. Measuring developmental deficit in children born at gestational age less than 26 weeks using a parent-completed developmental questionnaire. Acta Paediatr Int J Paediatr. 2006;95(11):1488–94.

149. Westerterp KR. Impacts of vigorous and non-vigorous activity on daily energy expenditure. Proc Nutr Soc. 2003;62:645–50.

150. Rasmussen KM, Yaktine AL. Weight gain during pregnancy: Reexamining the guidelines. Natl Acad Press. 2009;184(3):463–9.
